# Stabilization of β-catenin promotes melanocyte specification at the expense of the Schwann cell lineage

**DOI:** 10.1242/dev.194407

**Published:** 2022-01-24

**Authors:** Sophie Colombo, Valérie Petit, Roselyne Y. Wagner, Delphine Champeval, Ichiro Yajima, Franck Gesbert, Zackie Aktary, Irwin Davidson, Véronique Delmas, Lionel Larue

**Affiliations:** 1 Institut Curie, PSL Research University, INSERM U1021, Normal and Pathological Development of Melanocytes, Orsay, France; 2 Univ Paris-Sud, Univ Paris-Saclay, CNRS UMR 3347, Orsay, France; 3 Equipes Labellisées Ligue Contre le Cancer; 4 Department of Functional Genomics and Cancer, Institut de Génétique et de Biologie Moléculaire et Cellulaire, CNRS/INSERM/UNISTRA, 1 Rue Laurent Fries, 67404 Illkirch Cedex, France

**Keywords:** Pigmentation, Cell fate, Determination, Proliferation, Mitf, FoxD3, Mouse

## Abstract

The canonical Wnt/β-catenin pathway governs a multitude of developmental processes in various cell lineages, including the melanocyte lineage. Indeed, β-catenin regulates transcription of Mitf-M, the master regulator of this lineage. The first wave of melanocytes to colonize the skin is directly derived from neural crest cells, whereas the second wave of melanocytes is derived from Schwann cell precursors (SCPs). We investigated the influence of β-catenin in the development of melanocytes of the first and second waves by generating mice expressing a constitutively active form of β-catenin in cells expressing tyrosinase. Constitutive activation of β-catenin did not affect the development of truncal melanoblasts but led to marked hyperpigmentation of the paws. By activating β-catenin at various stages of development (E8.5-E11.5), we showed that the activation of β-catenin in bipotent SCPs favored melanoblast specification at the expense of Schwann cells in the limbs within a specific temporal window. Furthermore, *in vitro* hyperactivation of the Wnt/β-catenin pathway, which is required for melanocyte development, induces activation of Mitf-M, in turn repressing FoxD3 expression. In conclusion, β-catenin overexpression promotes SCP cell fate decisions towards the melanocyte lineage.

## Abstract (precis)

**Summary:** Activation of β-catenin in bipotent Schwann cell precursors during a specific developmental window induces Mitf and represses FoxD3 to promote melanoblast cell fate at the expense of Schwann cells in limbs.

## INTRODUCTION

Multipotent neural-crest cells (NCCs) in vertebrates constitute a transient population of cells arising from the dorsal part of the neural tube (Le Douarin and Kalcheim, 1999) that gives rise to numerous derivatives, such as neuronal and glial cells of the peripheral nervous system (PNS), smooth muscle cells and melanocytes. Melanocytes produce melanin, a tyrosine-based polymer, in specialized organelles, the melanosomes. Classical melanocytes are pigmented cells, which (1) are found in the skin (dermis or epidermis), (2) are involved in skin pigmentation and (3) are differentiated from melanoblasts derived from late-migrating NCCs that have followed the dorsolateral migratory pathway between the dermamyotome and the overlying ectoderm. These melanoblasts, referred to as first-wave melanoblasts, are specified as early as embryonic day (E) 8.5, before they start migrating along the dorsolateral pathway at E10.5 (Petit and Larue, 2016). Between E11.5 and E13.5, most melanoblasts enter the epidermis, where they actively proliferate (Luciani et al., 2011). Between E15.5 and E17.5, epidermal melanoblasts migrate towards the forming hair follicles. In the furry parts of adult mice, most melanocytes are found in the hair matrix, whereas only few interfollicular melanocytes remain in the epidermis after birth (Hirobe, 1984). Epidermal melanocytes are abundant in the hairless parts of the body, such as the tail and paws (Silvers, 1979), except in the palms and soles, which have very few (Kunisada et al., 1998; Fig. S1). Melanocytes are considered to be non-classical if they are found in organs other than skin, not involved in skin pigmentation, and/or have not followed the dorsolateral migratory pathway during development (Colombo et al., 2011). However, two types of non-classical melanocytes involved in skin pigmentation have been found, although they did not follow the dorsolateral migratory route. One corresponds to a population of cells originating around the time of gastrulation, most likely within the mesoderm, and ultimately residing within the dermis (Kinsler and Larue, 2018). These melanoblasts are referred to as ‘mesodermal-wave melanoblasts’. The other is derived from Schwann cell precursors (SCPs) and is referred to as second-wave melanoblasts. SCPs are multipotent embryonic progenitors covering all developing peripheral nerves and originate from early ventrally migrating NCCs (Furlan and Adameyko, 2018). Previous studies have shown that a significant number of melanocytes in the skin of the trunk and limbs are produced from SCPs adjacent to the spinal nerves that innervate the skin during development. Additionally, it has been shown that the glial versus melanocyte fate is highly dependent on nerve contact (Adameyko et al., 2009). The authors showed that SCP-derived melanoblasts migrating ventrally from the dorsal root ganglia are specified around E11 in the mouse. Although multiple elegant experiments had shown that the melanocytes and Schwann cells share a common glial-melanogenic bipotent precursor and can be transdifferentiated into each other *in vitro* (Dupin et al., 2000, 2003; Nitzan et al., 2013b; Real et al., 2006), the factors controlling the cell fate decisions between these two lineages remained unclear. More recent experiments started elucidating the molecular pathways involved in the glial-melanocyte switch. Those bipotent progenitors express various proteins, including Sox2, Sox9, Sox10, Fabp, Mitf, Pax3 and FoxD3 (Adameyko and Lallemend, 2010). It has been shown that FoxD3 represses the expression of *mitfa* in zebrafish (Curran et al., 2009), in melanoma cell lines and in cultured quail neural crest (Abel et al., 2013; Thomas and Erickson, 2009). Moreover, the downregulation of *FoxD3* is necessary for SCPs to follow a melanocyte fate (Adameyko et al., 2012; Jacob, 2015; Nitzan et al., 2013b).

β-Catenin plays crucial roles in multiple developmental processes, such as proliferation and cell fate decisions, owing to its dual function in cadherin-dependent cell-cell interactions and as a central component of the canonical Wnt signaling pathway (Aktary et al., 2016; Steinhart and Angers, 2018). Gain-of-function studies have shown induction of cellular proliferation of a number of cell types in transgenic mice expressing stabilized β-catenin (Gat et al., 1998; Imbert et al., 2001; Romagnolo et al., 1999). This pathway influences early melanoblast development, mainly through various common β-catenin/LEF targets, including Myc and Ccnd1, and a major downstream target of β-catenin in the melanocyte lineage, the Mitf-M transcription factor (Luciani et al., 2011). Mitf-M exerts survival and proliferation functions during the expansion of melanoblasts from the neural crest (Carreira et al., 2006; Hornyak et al., 2001) and regulates melanocyte differentiation by inducing the key enzymes of melanogenesis Tyr, Tyrp1 and Dct (Steingrimsson et al., 2004). The deletion of β-catenin specifically in migrating melanoblasts leads to hypoproliferation due to reduced Mitf-M expression (Luciani et al., 2011). Both the temporal and spatial fine-tuning of β-catenin and Mitf-M levels is required to regulate their various downstream targets and generate the required number of melanoblasts at the correct location during development. Apart from its role in neural crest induction and expansion, the Wnt/β-catenin signaling pathway has been implicated in neural crest cell fate decisions. Mice deficient for both *Wnt1* and *Wnt3a* exhibit a marked deficiency of Dct-positive neural crest-derived melanoblasts (Ikeya et al., 1997). β-Catenin has also been directly associated with melanoblast cell fate specification in various species using β-catenin gain- and loss-of-function approaches. In zebrafish, injection of *β-catenin* mRNA into a subpopulation of migrating NCCs induces the formation of pigmented cells (Dorsky et al., 1998). In mice, the conditional ablation of β-catenin in premigratory NCCs leads to a loss of melanocytes and sensory neurons (Hari et al., 2012), whereas its activation promotes the formation of the sensory neuronal lineage at the expense of other neural crest derivatives (Lee et al., 2004). A change in cell fate specification, rather than a proliferation defect, underlies the loss of melanocytes. Moreover, the expression of a constitutive activated form of β-catenin in bipotent cardiac neural crest cells, known to produce mainly smooth muscle cells and few melanocytes, promotes the melanocyte fate at the expense of the smooth muscle fate in the ductus arteriosus of embryonic hearts, leading to patent ductus arteriosus, a congenital disease (Yajima et al., 2013). Overall, these results demonstrate the essential role of the Wnt/β-catenin pathway in NCC and melanocyte fate determination.

We investigated the influence of β-catenin on the first and second wave of melanocyte development. A genetic approach was used in mouse to express a conditional mutant of β-catenin (βcat^Δex3^), known to be hyperactive (Harada et al., 1999), at specific times and in specific neural crest cell derivatives using either constitutive or inducible Cre lines under the control of the tyrosinase (Tyr) promoter (Delmas et al., 2003; Yajima et al., 2006). It has already been shown that the use of the Tyr::Cre transgene to conditionally delete specific genes targets the melanocytic lineage but also the enteric nervous system, smooth muscle cells in the heart, and the Schwann cell lineage (Puig et al., 2009; Radu et al., 2019; Yajima et al., 2013). We observed that constitutive activation of β-catenin led to hyperpigmentation of the paws as a result of promotion of the melanocyte fate at the expense of the glial fate at the time of SCP specification. At the molecular level, we show that β-catenin overexpression represses *FoxD3* expression through *Mitf*, thereby allowing SCPs to follow a melanocyte fate.

## RESULTS

### Constitutively active β-catenin (βcat^Δex3^) induces hyperpigmentation of the paws

**Fig. 1.**
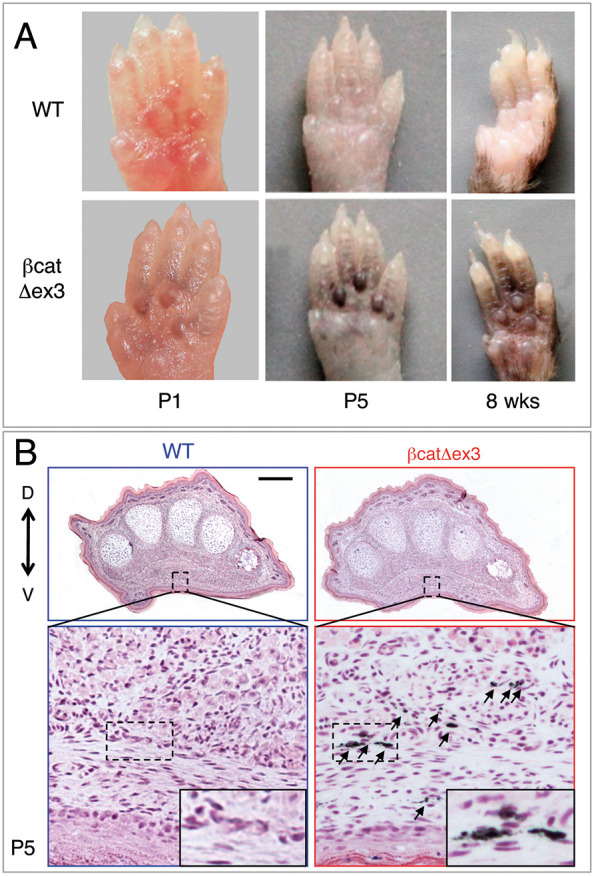
**Tyr::Cre/°; βcatex3^flox/+^ mice present palmoplantar hyperpigmentation. (**A) Ventral views of WT and βcat^Δex3^ anterior mouse paws in newborns (P1 and P5) and adults. (B) Hematoxylin and Eosin staining of P5 transversal paw sections. D, dorsal; V, ventral. Arrows point to pigmented cells. Insets show magnifications of the boxed areas. WT: °/°; βcatex3^flox/+^ or Tyr::Cre; βcatex3^+/+^. βcat^Δex3^: Tyr::Cre/°; βcatex3^flox/+^. Scale bars: 250 μm.

On a C57BL/6 background, we generated mice producing a constitutively active form of β-catenin (Tyr::Cre/°; βcatex3^flox/+^, hereafter βcat^Δex3^) in cells of the Tyr::Cre lineage by crossing Tyr::CreA mice (Delmas et al., 2003) with mice harboring a floxed exon 3 of β-catenin (Harada et al., 1999; Yajima et al., 2013). βcat^Δex3^ mutant mice displayed strong hyperpigmentation of the palms and soles with full penetrance (Fig. 1A, Fig. S2A). However, we did not observe strong hyperpigmentation on the back of the paws (Fig. S2A). Palmoplantar hyperpigmentation was already present at birth and was particularly striking at postnatal day (P) 5 (Fig. 1A). Transversal sections at the metatarsal level of paws from P1 and P5 newborn mice revealed high levels of pigmentation on the ventral side of the βcat^Δex3^ mutant paws, whereas it was absent from the wild-type (WT) paws (Fig. 1B, Fig. S2B). Moreover, this pigmentation was localized in the dermis, directly under the epidermis, as well as more deeply in the palmoplantar mesenchyme.

### β-Catenin is properly defloxed and activated in βcat^Δex3^ melanoblasts and melanocytes

The transcriptional activity of βcat^Δex3^ was previously assessed with the ‘TOP and FOP’ flash luciferase reporter assay and was shown to be five times higher than that of WT β-catenin (Yajima et al., 2013). Deletion of exon3 in βcat^Δex3^ mice was verified by PCR on genomic DNA extracted from mouse tails containing melanocytes (Fig. S3A,B). We verified the presence of β-catenin in the nucleus, a marker of its stabilization/activation, by immunofluorescence of skin sections during development and after the birth of Tyr::Cre/°; Dct::*lacZ* (WT-*lacZ*) and Tyr::Cre/°; βcat^ex3flox/+^; Dct::*lacZ* (βcat^Δex3^-*lacZ*) mice using β-galactosidase expression as a melanoblast/melanocyte marker (MacKenzie et al., 1997; Yajima et al., 2013). β-Catenin was present in the nucleus of βcat^Δex3^ melanoblasts in the epidermis of E14.5 embryos whereas it was localized at the membrane in WT mice (Fig. S3C). These results show that β-catenin was properly defloxed and activated in βcat^Δex3^ melanoblasts and melanocytes.

### The βcat^Δex3^ mutation does not affect coat color or truncal melanoblast proliferation

βcat^Δex3^ mutant mice have no distinctive coat color, ear or tail phenotype (Fig. S4A). The mutation of β-catenin is induced around E9.0, as the Tyr::Cre transgene begins to be expressed, after dorsolaterally migrating melanoblasts have been established. We evaluated the number of WT-*lacZ* and βcat^Δex3^-*lacZ* melanoblasts in the truncal region of E13.5-E18.5 embryos. From E13.5 to E15.5, the number of melanoblasts was determined on whole-mount embryos stained with X-gal in a region localized between the fore- and hindlimbs (ranging from approximately somite 13 to somite 25). There was no significant difference in melanoblast numbers at these stages between WT and mutant embryos (Fig. S4B). At E16.5 and E18.5, truncal melanoblasts were counted on embryo sections immunostained for β-galactosidase (Fig. S4C). Few or no melanoblasts were present in the dermis at these stages, as previously described for WT embryos (Luciani et al., 2011). The presented figures correspond to epidermal and hair-follicle melanoblasts. At E16.5, hair follicles have just initiated invagination from the epidermis whereas at E18.5 they extend into the dermis and numerous melanoblasts can be found entering and within the hair follicles. There was no difference in melanoblast numbers between WT and mutant mice at these two stages (Fig. S4C). We also investigated melanoblast proliferation in the skin of the trunk using bromodeoxyuridine (BrdU) incorporation assays on embryos collected at E16.5 and E18.5. There was no significant difference in the percentage of BrdU-positive melanoblasts at these stages (Fig. S4D). Overall, these results show that hyperactivation of β-catenin does not influence the development of already committed dorsolaterally migrating melanoblasts.

### Hyperpigmentation of βcat^Δex3^ ventral paws is due to an elevated number of melanocytes

**Fig. 2.**
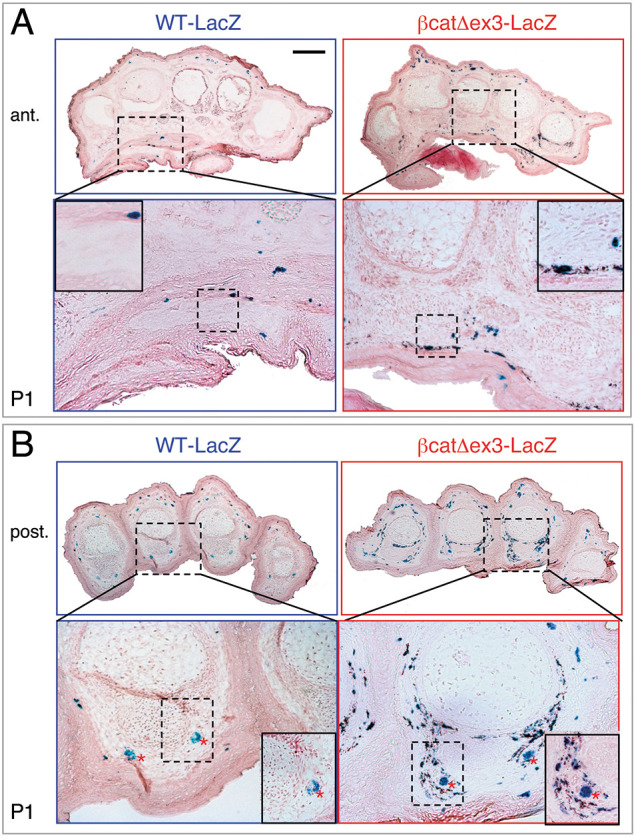
**Overexpression of an active form of β-catenin induces hyperpigmented Dct-positive cells on the ventral side of the paws.** (A,B) WT-*lacZ* and βcat^Δex3^-*lacZ* P1 paws were transversally sectioned, and stained with X-gal and Eosin. Anterior (ant.; A) and posterior (post.; B) paws at the metacarpal and phalangeal levels, respectively, are shown. In the dermis, mutant paws display high numbers of Dct-positive cells (melanocytes stained in blue, directly under the dermo-epidermal junction of the palmoplantar side of the paws (A) and around the bones of the digits (B). Note that these cells show a high accumulation of melanin. In the dermis, WT paws contain a very low number of Dct-positive cells or pigmentation. Note that some nerves are stained in blue in the posterior paws (red asterisks) in WT and mutant paws. Insets show magnifications of the boxed areas. WT-*lacZ*: °/°; βcatex3^flox/+^; Dct::*lacZ*/°. βcat^Δex3^-*lacZ*: Tyr::Cre/°; βcatex3^flox/+^; Dct::*lacZ*/°. Scale bars: 200 μm.

**Fig. 3.**
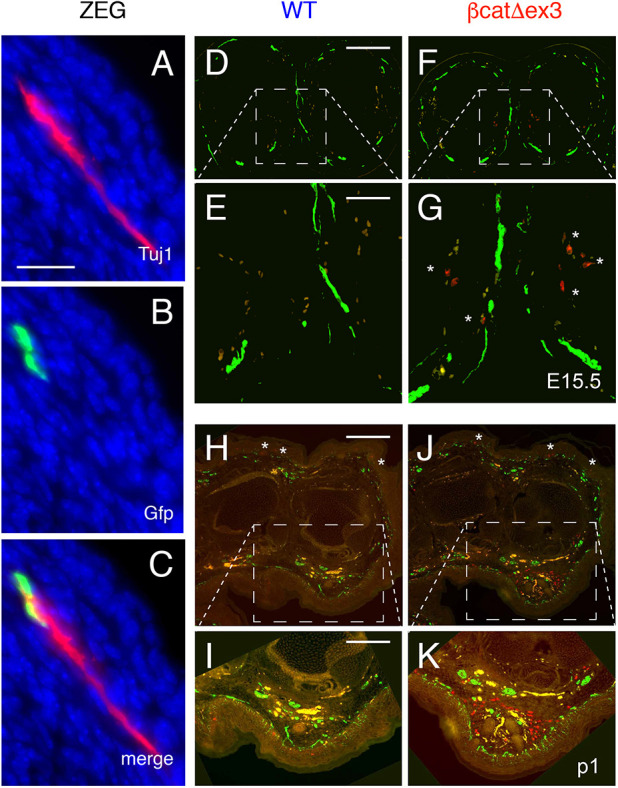
**GFP- and Pmel-positive cells are associated with Tuj1-positive cells during embryonic development.** (A-C) Forelimb of a Tyr::Cre/° ; ZEG/° (ZEG) E14.5 embryo was immunostained with Tuj1 (A, red) and GFP (B, green) antibodies, and counterstained with DAPI. Merge is presented in C. (D-K) WT (D,E,H,I) and βcat^Δex3^ (F,G,J,K) E15.5 (D-G) and P1 (H-K) paws were transversally sectioned, and immunostained for Pmel (red) and Tuj1 (green). The yellow staining corresponds to red blood cells (non-specific labeling). Asterisks highlight the presence of melanocytes in G,H,J. Note that some Tuj1-positive cells are close to Pmel-positive cells. WT: °/°; βcatex3^flox/+^. βcat^Δex3^: Tyr::Cre/°; βcatex3^flox/+^. Scale bars: 20 µm (A-C); 200 µm (D,F,H,J); 60 µm (E,G); 100 µm (I,K).

X-gal staining of transversal sections of P1 WT-*lacZ* and βcat^Δex3^-*lacZ* paws revealed numerous Dct-positive cells colocalized with strong pigmentation in the mutant palms and soles, whereas they were absent in WT littermates (Fig. 2A,B). X-gal staining also labeled the nerves in the posterior paws (Fig. 2B), but not in the anterior paws (Fig. 2A) (MacKenzie et al., 1997). These nerve-associated Dct::*lacZ*-positive cells were most likely melanoblasts and/or bipotent SCP and not nerve projections, as we observed a similar pattern of Dct-positive cells colocalized with pigment in both the anterior and posterior paws. The pigmentation pattern in mutant paws was located around the nerves, most likely following nerve projections (Fig. 2B). In the phalanges, pigmentation was strikingly localized around the bones of the digits (Fig. 2B), whereas at the metacarpal/metatarsal level it was mostly localized under the epidermis (Fig. 2A). We followed the expression of GFP (green fluorescent protein; corresponding to the cells that were defloxed by Cre) and Tuj1 (used as a marker for neuronal cells; also known as Tubb3) in Tyr::Cre/°;ZEG/° E14.5 embryos. We observed that some GFP- and Tuj1-positive cells were close to one to another in the ventral part of the limbs. This double labeling revealed that defloxed cells are close to neurons (Fig. 3A-C). Moreover, we performed staining of Pmel and Tuj1 in paw transversal sections and observed that some Pmel-positive cells were in close proximity to Tuj1-positive cells (Fig. 3D-K). These results suggest that ectopic melanocytes are present in the mutant paws.

### Hyperpigmentation of βcat^Δex3^ ventral paws is due to abnormal invasion of melanoblasts during development

**Fig. 4.**
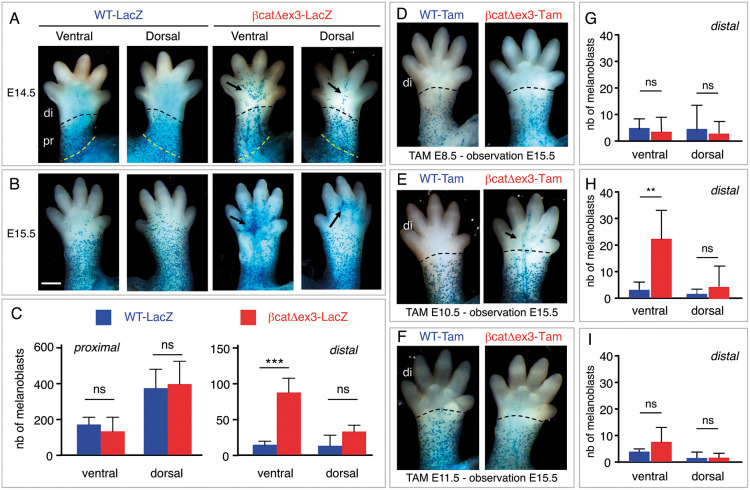
**β-Catenin favors the specification of SCPs towards melanoblasts.** (A-C) The number of melanoblasts is higher on the ventral side of the distal limbs of βcat^Δex3^ than WT mice. WT-*lacZ* and βcat^Δex3^-*lacZ* E14.5 (A) and E15.5 (B) paws were X-gal stained. Dorsal and ventral views are shown. The number of melanoblasts were estimated at E14.5 (C) in the distal (di) and proximal (pr) region of the limbs (delineated by the dashed lines in A). Arrows highlight ectopic melanoblasts. WT-*lacZ*: °/°; βcatex3^flox/+^; Dct::*lacZ*/°. βcat^Δex3^-*lacZ*: Tyr::Cre/°; βcatex3^flox/+^; Dct::*lacZ*/°. (D-I) Melanoblast numbers in the paws are increased when β-catenin is activated at E10.5. Ventral views of X-gal-stained WT (WT-Tam) and βcat^Δex3^ (βcat^Δex3^-Tam) E15.5 paws induced with tamoxifen at E8.5 (D), E10.5 (E) and E11.5 (F). The number of melanoblasts was estimated at E15.5 in the distal (di) part of the paw (delineated by the dashed lines in D-F) after tamoxifen induction at E8.5 (G), E10.5 (H) and E11.5 (I). Arrow in E highlights ectopic melanoblasts. No X-gal-positive cells were observed at E15.5 when Tam induction was performed at E12.5. WT-Tam: °/°; βcatex3^flox/+^; Dct::*lacZ*/°. βcat^Δex3^-Tam: Tyr::Cre-ER^T2^/°; βcatex3^flox/+^; Dct::*lacZ*/°. We examined a minimum of eight limbs for each situation. ***P<0.001; **P<0.01; ns, not significant (unpaired *t*-test). Scale bars: 5 mm.

The βcat^Δex3^ paw phenotype was already visible at birth, when the mice are normally unpigmented. It is thus likely the consequence of altered developmental processes. We analyzed the location and number of melanoblasts in E13.5 limbs and paws, when melanoblasts have started their migration to the limbs but have not yet reached the paws. There was no difference in melanoblast distribution between WT and mutant embryos at this stage (Fig. S5A). A difference started to appear at E14.5. Anterior mutant paws displayed melanoblasts ventrally in the palms, as well as few melanoblasts dorsally, whereas they were not present or were present only in very low numbers in WT embryos (Fig. 4A, Fig. S5B). There was a statistically significant increase in the number of Dct-positive melanoblasts in the distal region of the ventral limbs, but not in the proximal region of the limb (Fig. 4C). Although there was a tendency to increased numbers also on the dorsal side of mutant paws, the difference was not statistically significant (Fig. 4C). No phenotype was yet visible in the posterior paws at this stage (not shown). At E15.5, the phenotype was clearly visible ventrally in mutant paws. Large numbers of melanoblasts were found in the palms and soles and proximal part of the digits, whereas they were mostly absent from the WT paws. Melanoblasts could also be seen in the digits on the dorsal side of the paws (Fig. 4B, Fig. S5C). In WT mice, a clear front of migration of melanoblasts was apparent at the junction between the limb and paw (Fig. 4A, Fig. S5, black dashed lines). In mutant mice, however, melanoblasts appeared to cross this junction and continue their migration into the palms, soles and digits. Altogether, these results suggest that constitutively active β-catenin during the establishment of the melanocyte lineage induces melanoblast colonization into the palms and soles.

### Melanocytes from the palms and soles originate from the second wave of melanoblasts

**Fig. 5.**
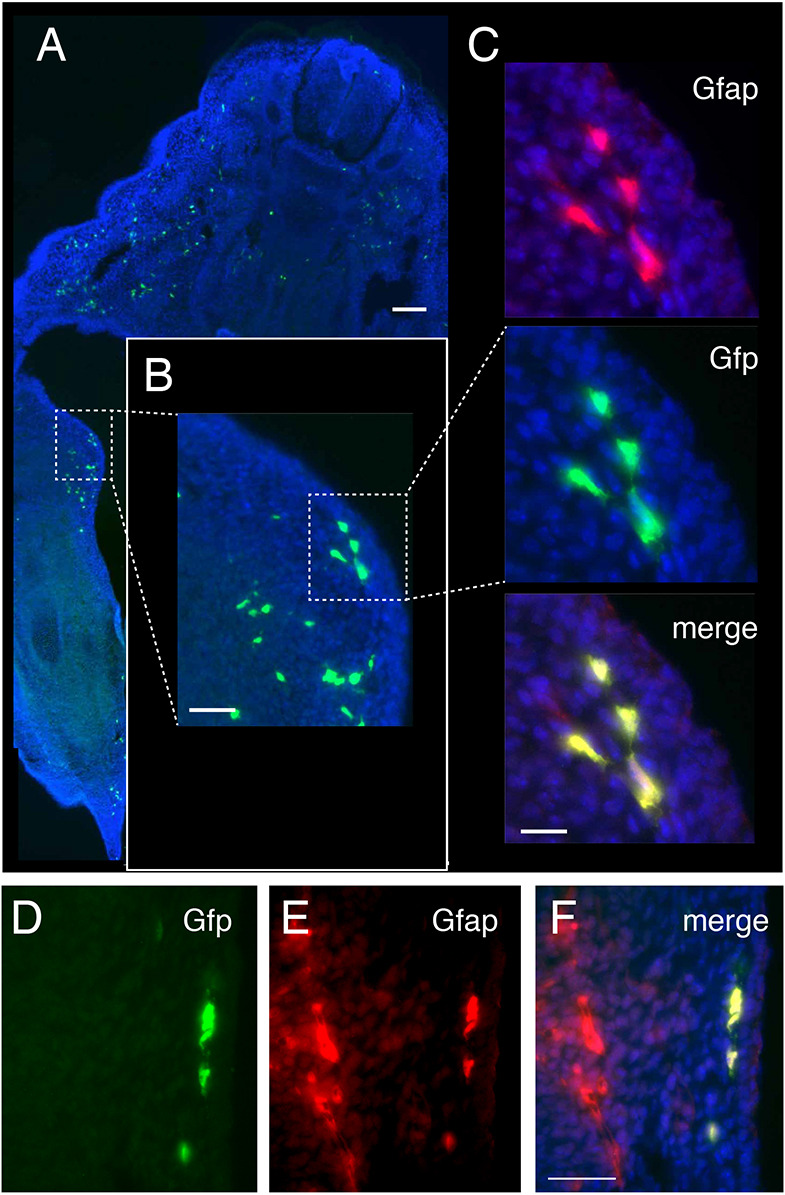
**GFAP-positive cells are defloxed in Tyr::Cre/°; ZEG/° mice during embryonic development.** (A) Transversal section at the level of the forelimb of an E14.5 Tyr::Cre/°; Z/EG embryo showing GFP expression (green) and counterstained with DAPI (blue). GFP is produced in cells that were defloxed by the Cre recombinase. To create this panel, several images of the same embryo section were taken separately and assembled using Adobe Photoshop. Enlargement of one zone is shown in B. (C) Proximal parts of the ventral part of forelimb of a Tyr::Cre/° ; ZEG/° embryo immunostained with Gfap (red) and GFP (green) antibodies, and counterstained with DAPI. (D-F) Distal parts of the ventral part of forelimb of a Tyr::Cre/° ; Z/EG embryo immunostained with GFP (green, D) and Gfap (red, E) antibodies, and counterstained with DAPI. Merge is presented in F. Note that in D-F the majority of the defloxed cells (GFP-positive) present in the distal part of the forelimb are producing Gfap, but Gfap-positive cells can be either defloxed or not. Scale bars: 100 µm (A); 25 µm (B); 10 µm (C); 50 µm (D-F).

**Fig. 6.**
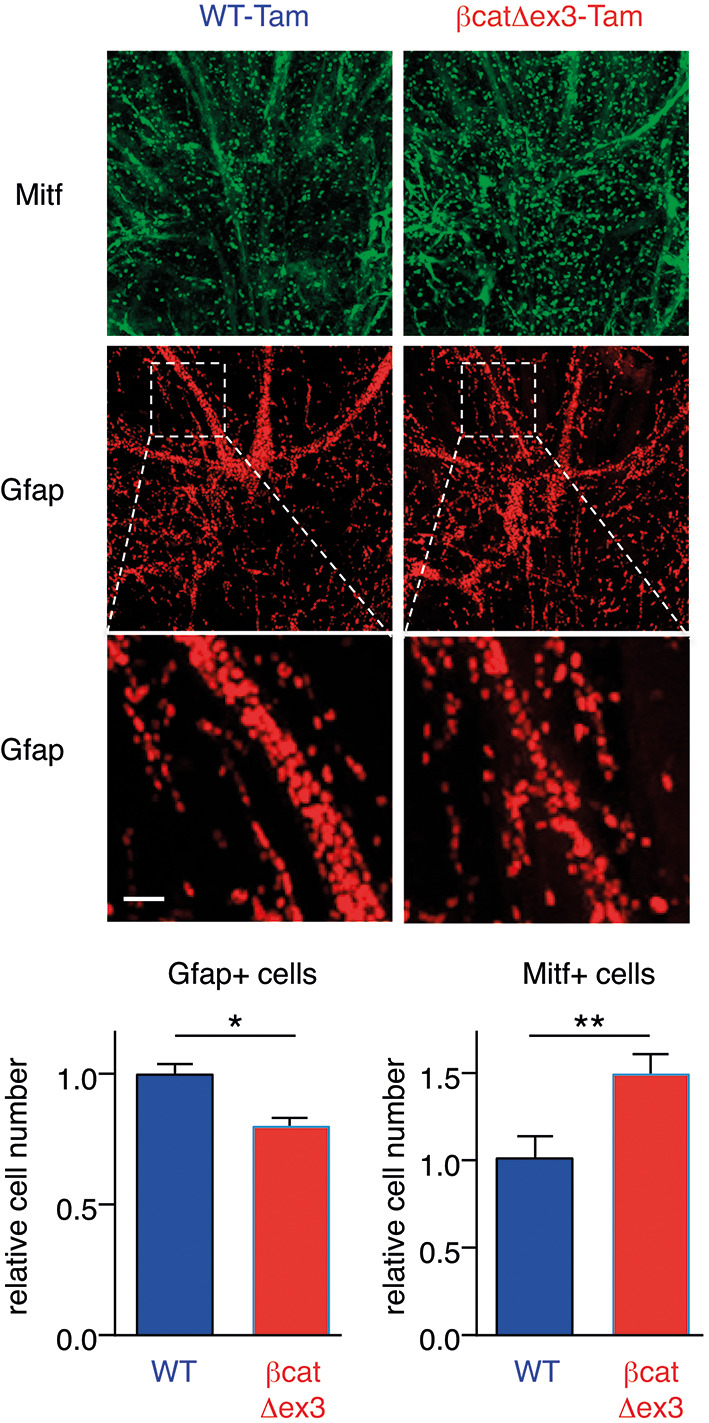
**The number of paw melanoblasts increases at the expense of glial cells when β-catenin is activated at E10.5.** Ventral views of WT-Tam and βcat^Δex3^-Tam E15.5 anterior paws from embryos induced with tamoxifen at E10.5 showing immunostaining of Mitf-M (green) and Gfap (red). Lower images show magnifications at the level of the nerve, highlighting the reduction of Gfap-positive cells in βcat^Δex3^ paws compared with WT. WT-Tam: °/°; βcatex3^flox/+^. βcat^Δex3^-Tam: Tyr::Cre-ER^T2^/°; βcatex3^flox/+^. The relative amounts of Gfap-positive and Mitf-positive cells are shown (WT versus βcat^Δex3^). Note that it is a 3D reconstruction; there is an accumulation of the signal over and below the nucleus. We followed six limbs for each situation. Statistical analysis was performed using an unpaired *t*-test. Error bars correspond to s.e.m. *P<0.05; **P<0.01. Scale bars: 50 μm.

Melanocytes are specified from the neural crest around E8.5-E9.0 (Le Douarin and Kalcheim, 1999), whereas they seem to specify from SCPs around E10.5-E11.5 (Fig. S6) (Adameyko et al., 2009; Van Raamsdonk and Deo, 2013). We used temporal induction of βcat^Δex3^ to reveal the origin of the melanoblasts invading the soles and palms and leading to the hyperpigmentation phenotype. We generated Tyr::CreER^T2^/°; βcatex3^flox/+^ mice (βcat^Δex3^-Tam), induced activated β-catenin at either E8.5, E10.5 or E11.5 with tamoxifen, and evaluated the location and number of melanoblasts in the distal part of the limbs at E15.5. The tamoxifen induction at E8.5 and E11.5 appeared to affect neither the number nor the localization of melanoblasts at E15.5 in the distal region of the ventral paws (Fig. 4D,F,G,I). However, tamoxifen induction at E10.5 resulted in a clear increase in the number of melanoblasts in the distal region of the ventral paws (Fig. 4E,H). These results suggested that bipotent SCPs may specify into melanocytes as early as E10.5 when the level of β-catenin is higher than normal. We followed the expression of GFP (corresponding to the cells that were defloxed) and Gfap (used as a marker for Schwann cells and Schwann cell precursors) in E14.5 embryo after Cre recombination of the ZEG transgene. We observed that cells are both GFP and Gfap positive in the ventral part of the limbs. This double labeling revealed that Gfap-positive cells expressed Cre or are derived from a cell that produced Cre under the control of the Tyr promoter (Fig. 5). We thus estimated the number of Schwann cells (Gfap-positive cells) and melanoblasts (Mitf-positive cells) in βcat^Δex3^ limbs. Expression of βcat^Δex3^ led to an increased number of melanoblasts and a decreased number of Schwann cells in the palms (Fig. 6). These results suggested that the expression of a constitutively active form of β-catenin in glial-melanogenic bipotent progenitors at the time of their fate determination promoted their differentiation into melanoblasts of the second wave at the expense of glial cells. Because SCPs are located along and migrate with axons of peripheral nerves, the ectopic melanocytes observed in the paws of βcat^Δex3^ mice would likely have migrated away from these nerves.

### β-Catenin induces the transcription of *Mitf* and the repression of FoxD3

**Fig. 7.**
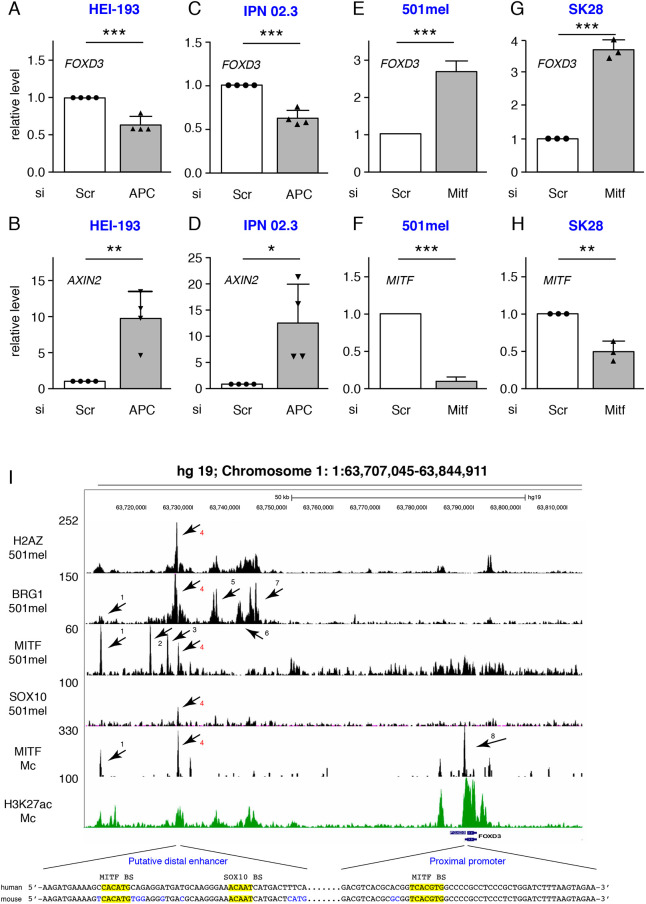
**MITF represses *FOXD3* expression.** (A-D) The relative amounts of *FOXD3* and *AXIN2* were determined by RT-qPCR from HEI-193 schwannoma and IPN 02.3 Schwann cell lines after siRNA-mediated knockdown of APC. (E-H) The relative amounts of *FOXD3* (E,G) and *MITF* (F,H) were determined by RT-qPCR in 501mel and SK28 human melanoma cell lines after siRNA-mediated knockdown of *MITF*. (I) UCSC screenshot of ChIP-seq data at the *FOXD3* locus. Shown are ChIP-seq data for H2AZ, BRG1 MITF and SOX10 in 501mel melanoma cells (GSE61967) and for H3K27ac (GSM958157) as previously described (Laurette et al., 2015). MITF ChIP-seq data in primary melanocytes (GSE50686) are from Webster et al. (2014). Binding sites are indicated by arrows in the proximal promoter in primary melanocytes (Mc) and in a putative distal enhancer in Mc and 501mel cells. The DNA sequences under the peaks are shown along with the syntenic regions from mouse. MITF- and SOX10-binding sites (BS) are highlighted in yellow. Each of these BS are bound by BRG1 and H2AZ, which are additional marks of regulatory sequences. Statistical analysis was performed using unpaired *t*-test. Error bars correspond to s.d. *P<0.05; **P<0.01; ***P<0.001.

The downregulation of *FoxD3* in SCPs is necessary to allow emergence of melanocyte cells (Adameyko et al., 2012; Jacob, 2015; Nitzan et al., 2013a) prompting us to investigate whether activation of β-catenin signaling affects *FoxD3* expression. Constitutive activation of β-catenin signaling by knocking down *APC* using an siRNA in HEI-193 human schwannoma (Fraenzer et al., 2003) and IPN 02.3 Schwann (Li et al., 2016) cell lines resulted in a significant decrease of *FOXD3* mRNA level compared with control scrambled siRNA (siScr) transfected cells (Fig. 7A,C). As a control, we showed that under the same conditions the levels of *AXIN2* mRNA, a well-known downstream target of β-catenin, was induced (Fig. 7B,D). It has previously been shown that *FOXD3* overexpression in melanoma cell lines or cultured quail neural crest cells resulted in repression of *MITF* expression (Abel et al., 2013; Thomas and Erickson, 2009). In a converse experiment, we show here that siRNA-mediated *MITF* silencing in 501mel and SK28 human melanoma cells leads to upregulation of *FOXD3* (Fig. 7E-H).

ChIP-seq in 501mel cells revealed that MITF occupied several sites at the *FOXD3* locus in a putative distal enhancer. There are at least four MITF bound sites (1-4) in 501Mel cells that are evident in this region (Fig. 7I). We focused on site 4 for several reasons. The MITF site is occupied in melanocytes, it is adjacent to a SOX10-bound site, it is associated with strong occupancy of BRG1 (SMARCA4) and H2AZ and marking by H3K27ac. All of these are hallmarks of active enhancer elements of melanoma cells. The requirement for SOX10 as a mark of the most functionally relevant sites was previously described (Laurette et al., 2015) and has been recently highlighted by other studies (Wouters et al., 2020). Nevertheless, MITF site 1 is also occupied in melanocytes and is associated with H3K27ac, but shows much lower BRG1 and H2AZ occupancy. Sites 2 and 3 are not occupied in melanocytes and show weak or no H3K27ac. We also note that sites 5-7 have all of the characteristics of regulatory elements with clear nucleosome-depleted regions. At each site, a consensus E-box sequence was present along with a SOX10-binding motif at the distal enhancer. In primary human melanocytes, MITF ChIP-seq identified a site in the proximal *FOXD3* (site 8) (Webster et al., 2014). Finally, these binding sequences were present at the otherwise well-conserved syntenic regions at the mouse *Foxd3* locus (Fig. 7I). Moreover, it is well accepted that H3K27ac is a mark of active enhancer elements and BRG1, by contrast, can also be associated with negative regulation (Laurette et al., 2015). *FOXD3* is not the only gene that is repressed by MITF. Indeed, numerous examples of genes (*PTEN*, *CDH1*, *GATA6*) are repressed by MITF (Berico et al., 2021; Dilshat et al., 2021; Hamm et al., 2021).

**Fig. 8.**
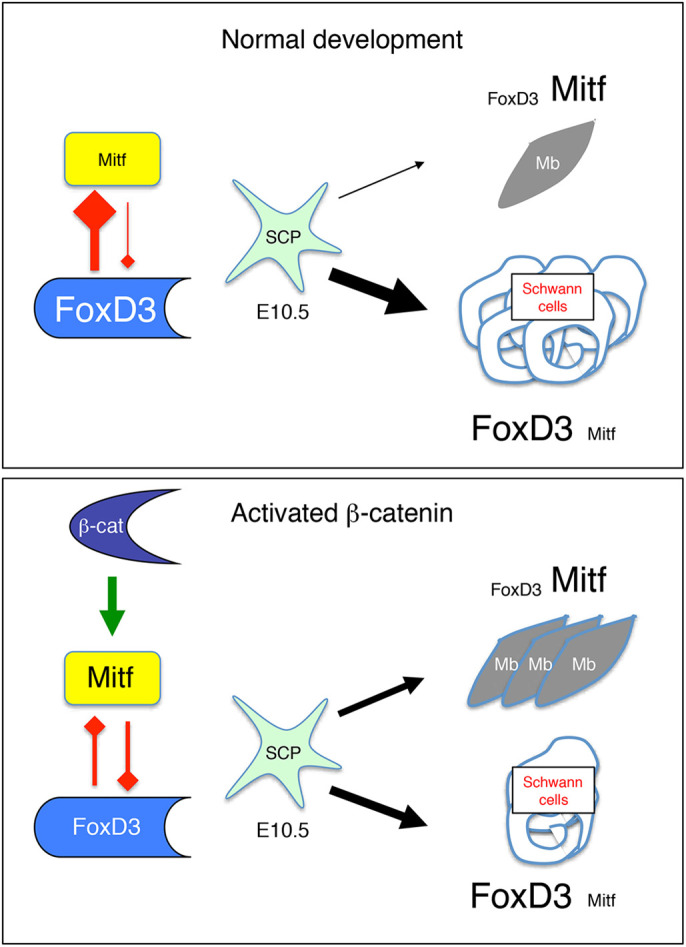
**Schematic of the determination of SCPs to generate Schwann cells and melanoblasts.** In this model, FoxD3 and Mitf regulate each other to specify Schwann cell and melanocyte lineage from SCPs around E10.5. In the presence of a high content of FoxD3 and low content of Mitf, SCPs would be directed towards the Schwann cell lineage and in the opposite conditions SCP would be directed towards the melanocyte lineage (Mb). In a situation in which β-catenin is activated, Mitf would be induced in SCPs to promote more efficiently the specification of melanocytes, and would also repress FoxD3 resulting in specification of fewer Schwann cells.

Taken together, these observations suggest the presence of a reciprocal regulatory feedback loop in the melanocytic lineage whereby FOXD3 represses *MITF* and MITF represses *FOXD3*. Given that the level of MITF expression and activity depends on numerous factors in the melanocytic lineage, this equilibrium may be rapidly shifted in favor of MITF when one of these molecular pathways, such as Wnt/β-catenin, is induced, leading to decreased FOXD3 levels and altered cell fate (Fig. 8).

## DISCUSSION

Here, we show that a constitutively active form of β-catenin (βcat^Δex3^) differentially affects melanoblast development in the trunk and paws. In the trunk region, expression of βcat^Δex3^ did not induce any major defects in developing melanoblasts, whereas it induced strong palmoplantar hyperpigmentation of the paws. This hyperpigmentation was due to the abnormal presence of melanocytes derived from the second wave of melanoblasts. Melanoblasts migrating in the palms and soles of the mutant mice were seen as early as E14.5, whereas they were mostly absent in WT mice. These results show that, once specified, βcat^Δex3^ does not influence the development of melanoblast of the first wave, but instead controls SCP cell fate decisions between glial and melanocyte lineages in the ventral migratory pathway. This difference could be due to a differential regulation of the endogenous Wnt signaling pathway in these two different environments. According to these results, the contribution of SCPs to melanocytes in the adult appeared to be restricted to the limbs.

It has been shown that Schwann cells and melanocytes are very close for several reasons besides the fact that they are derived from the neural crest. It has been shown that human melanocytes can transdifferentiate in Schwann cells (Chi et al., 2011) Spindle cell melanoma and pigmented neurofibroma possess cells with melanocyte and Schwann cell characteristics, with cells weakly producing Mitf and Tyr. This gives these cells the potential opportunity to perform a bidirectional differentiation (Motoi et al., 2005; Winnepenninckx et al., 2003). Melanotic schwannoma, a rare variant of nerve sheath tumors that arise from spinal nerve roots, is composed of neoplastic Schwann cells that produce melanin (Alexiev et al., 2018). The molecular status of these cells were not described, but we hypothesize that they produce Tyr and, therefore, Mitf. Moreover, Tyr promoter activity was detected in cells other than melanocytes, such as the cortex, olfactory system, hippocampus, epithalamus and substantia nigra, during embryonic development (Tief et al., 1998). Finally, adult melanocytes expressing Tyr have been detected in many tissues and organs where they would not be initially expected (for example, see reviews Brito and Kos, 2008; Colombo et al., 2011; Gudjohnsen et al., 2015; Yajima and Larue, 2008).

### Hyperpigmentation of the paws

Hyperpigmentation of the palms and soles had previously been described in humans and mice after cell non-autonomous induction. Human palmoplantar fibroblasts express the Wnt/β-catenin signaling inhibitor DKK1, which inhibits melanocyte function and growth by regulating β-catenin (Yamaguchi et al., 2004; Yamaguchi et al., 2008). Downregulation of β-catenin leads to inhibition of Mitf-M expression, and of its downstream target Tyr, the key enzyme of melanogenesis. Increasing β-catenin levels in SCP-derived melanocytes may counteract the effects of Dkk1 in palmoplantar skin, promoting melanocyte differentiation after inducing Mitf and Tyr. Overexpression of Kitl (Steel factor) in the basal layer of the epidermis in mice induces palmoplantar hyperpigmentation (Kunisada et al., 1998). The authors found melanoblasts in the footpads of E16.5 mutant embryos, whereas they were not present in WT littermates. As Kit signaling is involved in melanoblast migration, they proposed that increased Kit signals promote migration of melanoblasts throughout the entire paw epithelium. This explanation is certainly valid. However, Kit signaling in melanocytes indirectly regulates β-catenin, through the PI3K pathway, and Mitf-M, through the MAPK pathway. Thus, in keeping with the results obtained here, an alternative and/or complementary explanation for the palmoplantar hyperpigmentation is enhanced melanoblast specification from SCPs. Other mutations lead to hyperpigmentation not only of the paws but also in pinna, in tails of adult mice and in hair-bearing skin. A series of mutants are associated with G protein-coupled receptors such as Gnaq (Dsk1 – V179M, and Dsk10 – F335L) and Gna11 (Dsk7 – I63V). Gnaq and Gna11 are the main mediators of Ednrb, a key regulator of melanocyte proliferation and survival (Jain et al., 2020; Van Raamsdonk et al., 2004). Another series of mutants is associated with p53 (Trp53), Kitl, Kit, Rps19 (Dsk3 – T316A/Y54N) and Rps20 (Dsk4 – T29C/L32P and T2201A in 3′ UTR). Interestingly, heterozygous Rps19 or Rps20 mutation in keratinocytes activates p53, which induces the level of Kitl to induce Kit present at the surface of melanocytes (McGowan et al., 2008).

### Specification

As previously mentioned, β-catenin is involved in cell fate specification, a process involving complex combinations of cell intrinsic and extracellular signals that need to be correctly delivered in time and space. The role of β-catenin in the specification of first-wave melanocytes has been clearly demonstrated. The high level of β-catenin in premigratory neural crest cells promotes the expansion and differentiation of mouse melanoblasts as shown *in vitro* by Dunn and colleagues after infecting neural tube with RCAS-Wnt1 retrovirus (Dunn et al., 2000) or *in vivo* by Hari and colleagues after inducing β-catenin using the Wnt1-Cre mice (Hari et al., 2002). In both cases, the high level of β-catenin induces Mitf-M and Tyr, at a time when some cells are not yet determined. In both cases, Wnt1 is produced in premigratory neural crest cells, but Tyr is not. As a consequence, when using Tyr::CreERt2 with an early tamoxifen induction or Tyr::Cre mice, the expansion and differentiation of mouse melanoblasts from premigratory neural crest cells would not be possible, because Cre or CreERt2 are not present in these cells; the promotion of the first wave of melanoblasts may, therefore, not occur. It is also important to remember that it is still not known whether CreERT2 is produced at E8.5. Using Wnt1::Cre or Tyr::Cre mice to delete exon 3 of β-catenin, melanocytes were present at a higher level than normal in various part of the body, including sympathetic ganglia, spleen, heart and brain (Yajima et al., 2013). However, using Tyr::CreERt2 with a tamoxifen induction at E10.5, we did not observe melanocytes at ectopic sites other than the palms. The inactivation of β-catenin in NCCs prior to melanoblast specification using Wnt1::Cre shows that β-catenin is essential for the generation of melanoblasts. The absence of β-catenin apparently does not impair early SCP specification, as specific markers are produced (Hari et al., 2002). Thus, SCPs and second-wave melanocytes still form in these animals. This series of experiments showed the crucial function of Wnt signaling in driving early melanoblast specification and could explain the absence of first-wave melanocytes (i.e. those migrating dorsolaterally), but the importance of β-catenin in the generation of second-wave melanocytes was still unknown. As Schwann cells and second-wave melanocytes share a common SCP precursor, we hypothesized that β-catenin in the βcat^Δex3^ mutant mice is activated in SCPs that migrate via the ventral pathway, altering their fate and promoting their differentiation into melanocytes. Whereas neural progenitors and glial cells express the Foxd3 transcription factor, it is not expressed in melanoblasts (Kos et al., 2001). As Mitf is the key transcription factor specifying the melanocyte lineage and knowing that SCPs express Foxd3, Mitf and Sox10, it is likely that SCP fate is governed by the relative amounts/activities of Foxd3 and Mitf. In agreement with this hypothesis, constitutive activation of β-catenin in Schwannoma cells led to FOXD3 repression, whereas *MITF* silencing upregulated FOXD3 expression in melanoma cell lines. Moreover, MITF binds to regulatory elements at the *FOXD3* locus in human melanoma cells and primary melanocytes and may therefore directly inhibit its expression. In contrast, overexpression of FOXD3 in melanoma cell lines represses *MITF* expression (Abel et al., 2013; Thomas and Erickson, 2009). Together, these observations support the idea that a direct and reciprocal negative regulation of FOXD3 and MITF expression can affect SCP fate. This model is reminiscent of the reciprocal negative regulation seen with MITF and JUN that affects the phenotype switch between melanocytic and undifferentiated melanoma cell states (Riesenberg et al., 2015). Based on these observations, we propose that high β-catenin levels in SCP at the time of their specification increases *Mitf* expression, hence repressing *FoxD3* expression and enhancing melanocyte specification at the expense of glia. At this point, we cannot exclude the possibility that β-catenin might repress *FoxD3* expression through other pathways. Such a model is supported by the reduced numbers of Gfap-positive cells and increased numbers of Mitf-positive or Dct-positive cells observed in the paws of βcat^Δex3^ mice, suggesting that a cell fate switch occurred.

### Acral melanoma

Although the number of melanocytes in the soles of the feet and palms of the hands are very limited, these cells may transform in acral melanoma (ALM). ALM and nodular melanoma (NM) are more aggressive than superficial spreading melanoma (SSM). The prevalence of ALM is higher in Asians (50%) than in Caucasians (10%) (Bertrand et al., 2020; Bradford et al., 2009; Lee et al., 2012). This is because NM and SSM are very rare in Asians, but the risk to develop an ALM appears to be similar between Asians and Caucasians. At the molecular level, the main mutations in ALM and non-ALM are similar (they include mutations in the *BRAF*, *NRAS*, *NF1* and *KIT* genes) but the prevalence differs (Moon et al., 2018; Zebary et al., 2013). NM and SSM arise from melanocytes determined from the first wave of melanoblasts, and one could speculate that ALM arises from melanocytes derived from the second wave of melanoblasts. This hypothesis has to be put in perspective with a recent work showing that ALM may emerge from melanocyte stem cells located in sweat glands (Eshiba et al., 2021). Although sun exposure is a well-established cause for melanoma development, the soles and palms are non-sun-exposed regions, raising the issue of the importance of the embryonic origin of melanocytes in melanomagenesis and how this may influence their aggressiveness when transformed.

### Conclusion

β-Catenin appears to play a complex role in the melanocyte lineage, depending on tight regulation of its levels and time and place of induction. We show here that expression of βcat^Δex3^ after specification of the melanoblasts of the first wave in Tyr::Cre- and Tyr::CreER^T2^-expressing cells does not appear to affect melanoblast development in the dorsolateral pathway, but favors melanoblast specification in the ventral pathway.

## MATERIALS AND METHODS

### Transgenic mouse generation and genotyping

Animal care, use and experimental procedures were conducted in accordance with recommendations of the European Community (86/609/EEC) and Union (2010/63/UE) and the French National Committee (87/848). Animal care and use were approved by the ethics committee of the Curie Institute in compliance with institutional guidelines.

Mice with conditional constitutive stabilization of β-catenin were generated by mating Tyr::CreA and Tyr::Cre-ER^T2-Lar^ (designated in the text as Tyr::Cre-ER^T2^) transgenic mice (Delmas et al., 2003; Yajima et al., 2006) with animals homozygous for a floxed allele of β-catenin, with LoxP sites flanking exon 3 (Δex3) (Harada et al., 1999). Z/EG mice were used to follow defloxed cells (Novak et al., 2000). Transgenic mice were maintained on a pure C57BL/6J background (backcrossed at least ten times). All animals were housed in specific pathogen-free conditions in the animal facility. Mice were genotyped using DNA isolated from tail biopsies using standard PCR conditions. The Tyr::Cre transgene (0.4 kb fragment) was detected by PCR, as previously described (Delmas et al., 2003). For detection of the floxed (570 bp) and WT (376 bp) alleles of the β-catenin gene, PCR amplification was carried out with the forward primer (LL523) 5′-GACACCGCTGCGTGGACAATG-3′ and the reverse primer (LL524) 5′-GTGGCTGACAGCAGCTTTTCTG-3′. The forward primer (LL667) 5′-GTGGACAATGGCTACTCA-3′ and the reverse primer (LL668) 5′-CTGAGCCCTAGTCATTGCAT-3′ were used for detection of the WT (715 bp) and deleted (450 bp) alleles of the β-catenin gene. The PCR conditions were as follows: 5 min at 94°C followed by 35 cycles of 20 s at 94°C, 30 s at 56.5°C, 45 s at 72°C, and a final extension of 10 min at 72°C.

### Tamoxifen injection

Pregnant C57BL/6J mice were injected intraperitoneally at E8.5, E10.5 or E11.5 with 0.5 mg tamoxifen (Sigma-Aldrich) per 20 g body weight (diluted in corn oil). This dose of tamoxifen was not optimal but higher doses induced embryonic death and resorption of the embryos.

### Histology

Transgenic mice carrying mutations of interest were crossed with Dct::*lacZ* mice (MacKenzie et al., 1997) and the resulting embryos collected at various times during pregnancy. Embryos were stained with X-gal, as previously described (Delmas et al., 2003). Paws of newborn mice at P1 were dissected, washed in PBS, and fixed by incubation in 0.25% glutaraldehyde in PBS for 50 min at 4°C. They were then incubated in 30% sucrose in PBS overnight, followed by 30% sucrose/50% Optimal Cutting Temperature Compound (OCT) in PBS for 5 h and then embedded in OCT. Cryosections (7 µm thick) were stained either with Hematoxylin and Eosin or X-gal as follows: they were washed twice in PBS at 4°C, and incubated twice, for 10 min, in permeabilization solution (0.1 M phosphate buffer pH 7.3, 2 mM MgCl_2_, 0.01% sodium deoxycholate, 0.02% NP-40) at room temperature (RT). They were then incubated in staining solution (0.4 mg/ml 5-bromo-4-chloro-3-indolyl-D-galactosidase, 2 mM potassium ferricyanide, 2 mM potassium ferrocyanide, 4 mM MgCl_2_, 0.01% sodium deoxycholate and 0.02% NP-40 in PBS) overnight at 30°C. Sections were post-fixed in 4% paraformaldehyde (PFA) overnight at 4°C, washed in PBS, and stained with Eosin. Paws of newborn mice at P5 were fixed in 4% PFA, dehydrated, and embedded in paraffin by standard methods. Paraffin sections (7 µm thick) were stained with Eosin.

### Immunostaining

Embryos were collected at various stages of development. Embryos and/or skins dissected from the back of the mice were washed in PBS and fixed by overnight incubation in 4% PFA. They were then either incubated in 30% sucrose in PBS overnight, followed by 30% sucrose/50% OCT in PBS for 5 h and embedded in OCT, or dehydrated and embedded in paraffin. Sections (7 µm thick) were washed with PBS-Tween 0.1% (PBT) for 10 min. Antigens were then retrieved by incubation for 20 min in citric acid buffer (pH 7.4) at 90°C. Non-specific binding was blocked by incubation with 2% skimmed milk powder in PBT. Sections were incubated overnight at 4°C with various primary antibodies. Rabbit polyclonal antibody against β-catenin (Abcam ab6302, 1/1000), chicken polyclonal antibody against β-galactosidase (Abcam ab9361, 1/400), mouse monoclonal antibody against tubulin β3 (clone TUJ1, BioLegend 801213, 1/200), rabbit monoclonal antibody against gp100/Pmel (Abcam ab137078, 1/300) and rabbit polyclonal antibody against GFP (APR#06 Curie Platform, 1/300) were used. Sections were washed three times in PBT for 5 min each and incubated with secondary antibodies for 1 h at 37°C. The secondary antibodies used were donkey Alexa 488-anti-rabbit, donkey Alexa 555-anti-chicken, donkey Alexa 488-anti-mouse and donkey Alexa 555-anti-rabbit (Molecular Probes) each at a dilution of 1/500. Sections were incubated in DAPI for 10 min, washed three times in PBT, for 10 min each, and mounted in mounting media containing N-propylgalate. Conventional fluorescence photomicrographs were obtained with a Leica DM IRB inverted routine microscope.

### Whole-mount immunostaining

E13.5 and E15.5 embryos paws were collected and fixed in 4% PFA in PBS pH 7.5 (Euromedex) for 6 h prior washing them three times in PBT. Paws were dehydrated in a series of PBS/methanol incubations (25%, 50%, 75% and 100%) for 10 min each. Paws were incubated for 24 h in 100% methanol at 4°C prior to bleaching for 24 h in a 1:2 mixture of H_2_O_2_ and 30% methanol. Paws were washed three times in 100% methanol prior to post-fixation overnight in a 1:4 mixture of DMSO and pure methanol. Paws were sequentially rehydrated in PBS/methanol (75%, 50%, 25% and 0%) for 10 min each prior to washing them twice in PBT. Paws were incubated overnight at RT in PBS containing 5% donkey serum, 1% bovine serum albumin and 20% DMSO as blocking solution. After blocking, paws were incubated with primary antibody in the blocking solution at 1/1000 for 5 days at RT. Primary antibodies were rabbit anti-neurofilament (Abcam ab9034), mouse anti-Gfap (Sigma-Aldrich C9205 and Cell Signaling Technology 3670) and goat anti-Mitf (R&D Systems AF5769). Secondary antibodies were Alexa Fluor 555 (Invitrogen A-31572), Alexa Fluor 488 (Invitrogen A21202) and Alexa Fluor 633 (Invitrogen A-21082), diluted in blocking solution at 1/1000, and incubated overnight at RT. Staining was ended after incubation of the paws in DAPI for 4 h at RT. Embryos were dehydrated in PBS/methanol (25%, 50%, 75% and 100%) for 10 min each at RT. Chambers created using 1-mm-thick FastWells (Sigma-Aldrich) on a glass slide were used to mount the paws. Each paw was fixed on to the glass slide with 1% NuSieve Agarose (Sigma-Aldrich) and covered with methanol. After three washes with methanol, paws were incubated twice for 5 min with 50% methanol and 50% BABB (1:3 benzylalcohol and 2:3 benzylbenzoate, Sigma-Aldrich), and then three times in pure BABB for 5 min each (or until the sample is cleared). The chamber was closed with a coverslip and sealed with nail polish prior to examination under the microscope.

### Confocal imaging and ImageJ treatment for 3D reconstruction

*z*-sections were acquired every 5 µm for the dorsal and ventral part of the limb with a Confocal Leica SP5 microscope. Then, the plug-in PureDenoise was used on the stack to increase the signal, and finally the filter substrate background was used to remove the remaining background. 3D reconstructions were performed from stacks containing the same number of sections and the same biological structures in WT and mutants, using 3D project in ImageJ without interpolation.

### BrdU labeling

Melanocyte proliferation was analyzed using BrdU labeling *in vivo* on embryos at various stages of development. BrdU (100 µg/ml, BD Biosciences) was injected intraperitoneally into the pregnant mother 2 h before sacrifice, in the form of two 50 µg/ml injections administered at 20-min intervals. Embryos were collected for immunohistochemistry. They were fixed and stained, as described above, with mouse monoclonal anti-BrdU antibody (BD Biosciences 555627, 1/200) and chicken polyclonal anti-β-galactosidase antibody (Abcam ab9361, 1/400). Donkey Alexa 488-anti-mouse and donkey Alexa 555-anti-chicken (Molecular Probes) were used as secondary antibodies each at a dilution of 1/500.

### Melanoblast counts on the paws

Pictures of X-gal-stained paws were taken using a binocular magnifying glass with a 1× objective. Proximal area (from the body to the migrating front, between the dashed yellow and black lines) and distal area (after the black dashed line) were delimited on the image (Fig. 4). Blue dots (melanoblasts) were counted using ImageJ software. At least five embryos were counted for each genotype at each stage in both areas.

### Cell culture and siRNA-mediated knockdown

501mel and SK28 human melanoma cell lines were grown in RPMI 1640 media (Gibco) supplemented with 10% fetal calf serum (Gibco) and 1% Penicillin-Streptomycin (Gibco) (Rambow et al., 2015). HEI-193 Schwannoma and IPN 2.03 Schwann cells were grown in DMEM media (Gibco) supplemented with 10% fetal calf serum (Gibco) and 1% Penicillin-Streptomycin (Gibco) (Fraenzer et al., 2003; Li et al., 2016). Cells were maintained at 37°C in a humidified atmosphere containing 5% CO_2_. Cells were routinely tested for the absence of mycoplasmas using MycoAlert (Lonza). siRNA targeting human MITF (M-008674) and APC (L-003869) were purchased from Dharmacon. Si Scramble (siSCR), with no known human targets, was purchased from Eurofins Genomics. Cells were transfected with 100 pmol siRNA or siScr with Lipofectamine 2000 (Invitrogen) and assayed for mRNA expression at 48 h post-transfection.

### RNA extraction and RT-qPCR

Total RNA was extracted from cell lines using the miRNeasy kit (Qiagen). M-MLV reverse transcriptase (Invitrogen) was used according to the manufacturer's protocol to synthesize cDNA from 1 μg total RNA in combination with random hexamers. Quantitative RT-PCR was performed with the iTaq Universal SYBR Green Supermix (Bio-Rad) and the primers listed below, using a QuantStudio 5 thermocycler (Applied Biosystems) in a final reaction volume of 25 μl under the following conditions: 95°C for 1.5 min, 40 cycles of 95°C for 30 s, 60°C for 60 s, with a final melting curve analysis. Relative expression was determined by the comparative ΔΔCt method. PCR primers: FOXD3 forward: 5′-CATCCGCCACAACCTCTC-3′; FOXD3 reverse: 5′-CATATGAGCGCCGTCTG-3′; MITF forward: 5′-CTATGCTTACGCTTAACTCCA-3′; MITF reverse: 5′-TACATCATCCATCTGCATACAG-3′; AXIN2 forward: 5′-CCTAAAGGTCGTGTGTGGCT-3′; AXIN2 reverse: 5′-GTGCAAAGACATAGCCAGAACC-3′; TBP forward: 5′-CACGAACCACGGCACTGATT-3′; TBP reverse: 5′-TTTTCTTGCTGCCAGTCTGGAC-3′.

### Statistical analysis

Statistical tests are detailed in the figure legends. Data are presented as mean±s.e.m. Statistical analyses were performed with Prism 5 software (GraphPad).

## Supplementary Material

Supplementary information

Reviewer comments
