# Supplementary material for: Stabilization of β-catenin promotes melanocyte specification at the expense of the Schwann cell lineage
Source: Development. 2022 Jan 24;149(2):dev194407. doi: 10.1242/dev.194407 (PMC8917410; doi:10.1242/dev.194407)
Supplement: Supplementary information [file develop-149-194407-s1.pdf]

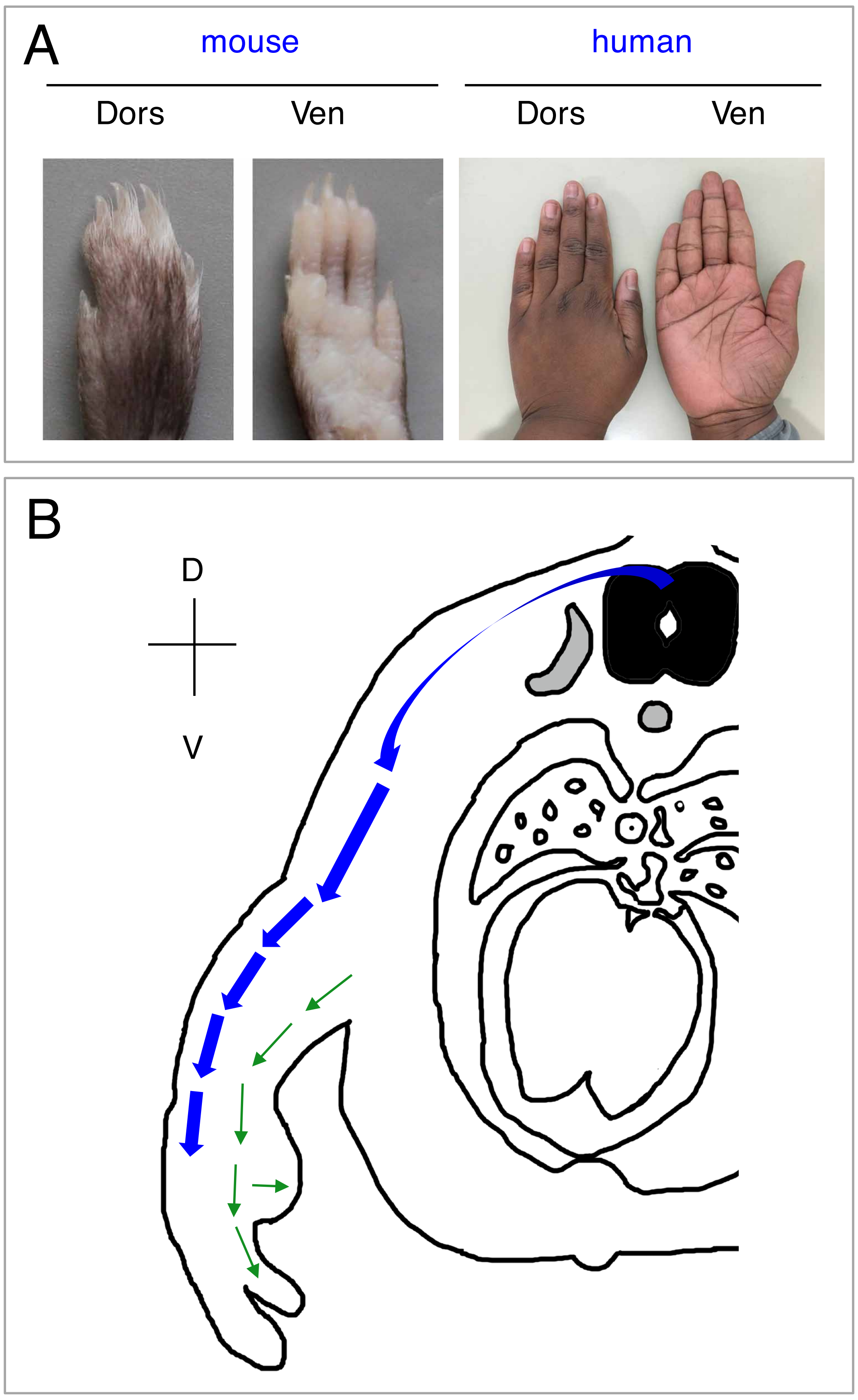


**Fig. S1. Human and mouse pigmentation of the limb extremities**
(A) Human and mouse palms are depigmented due to a low number of melanocytes. Back of the human and mouse extremities are pigmented. (B) Schematic of the migration of melanoblasts represented on a transversal section at the level of the limb. Blue arrows represent the migration path of the melanoblasts of the first wave (asymmetric and dorso-lateral). Green arrows represent the migration path of the melanoblasts of the second wave (asymmetric, dorso-ventral and derived from the Schwann cell precursors - SCP). D = dorsal. V = ventral.

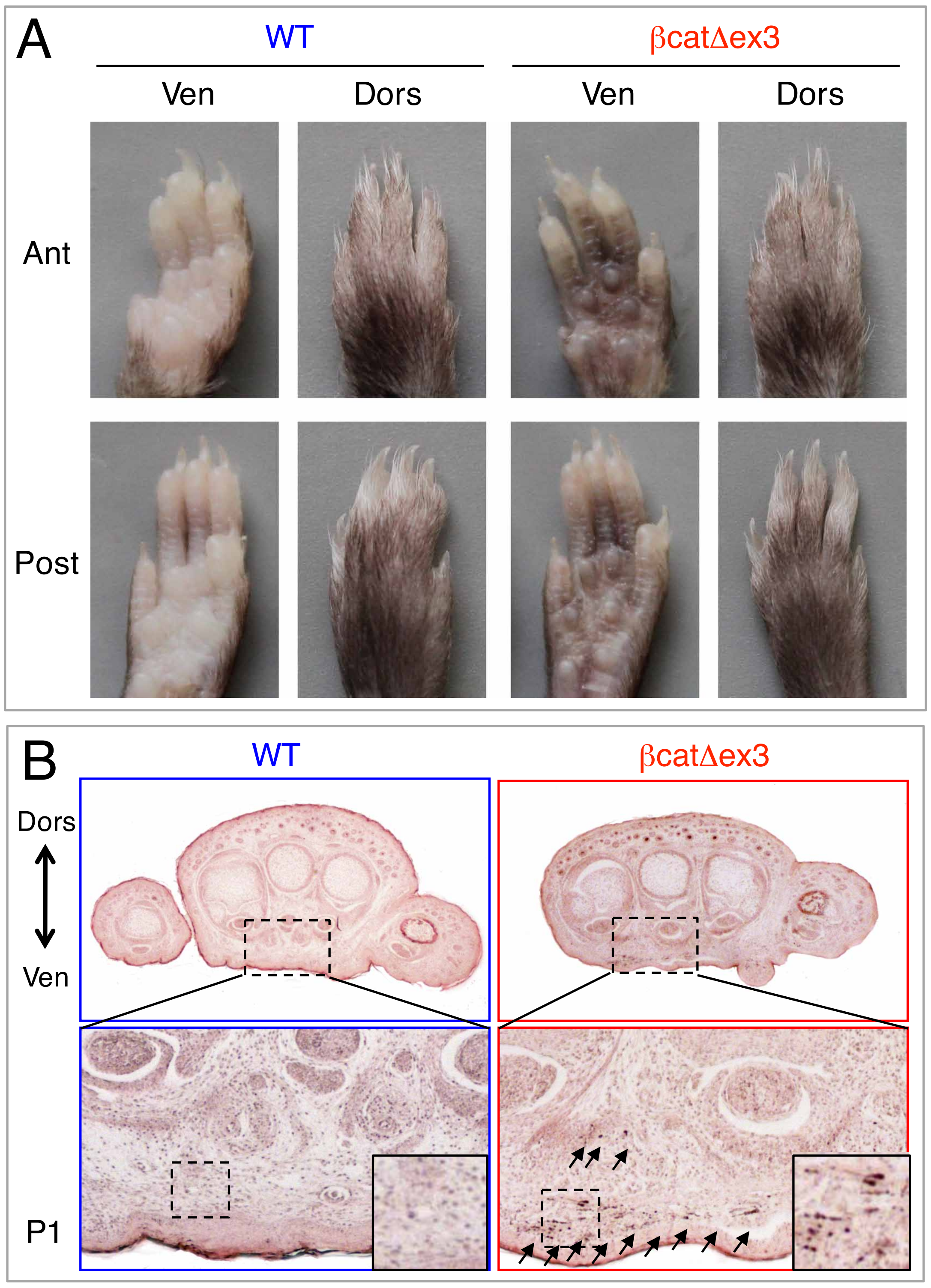


**Fig. S2. The expression of an active form of β-catenin induces an accumulation of pigment on the palmoplantar side of the paws.**
(A) Ventral (Ven) and dorsal (Dors) views of WT and βcatΔex3 anterior (Ant) and posterior (Post) paws in adult mice. Note that the hyperpigmentation on the dorsal side of the paws is rarely visible. (B) Eosin staining of P1 (postnatal day 1) transversal paw sections at the metatarsal level. Arrows are pointing at melanin pigment. Note that no pigment is observed in the WT counterpart. WT = (°/°; βcatex3flox/+) or (Tyr::Cre; βcatex3+/+). βcatΔex3 = (Tyr::Cre/°; βcatΔex3flox/+).

Development • Supplementary information

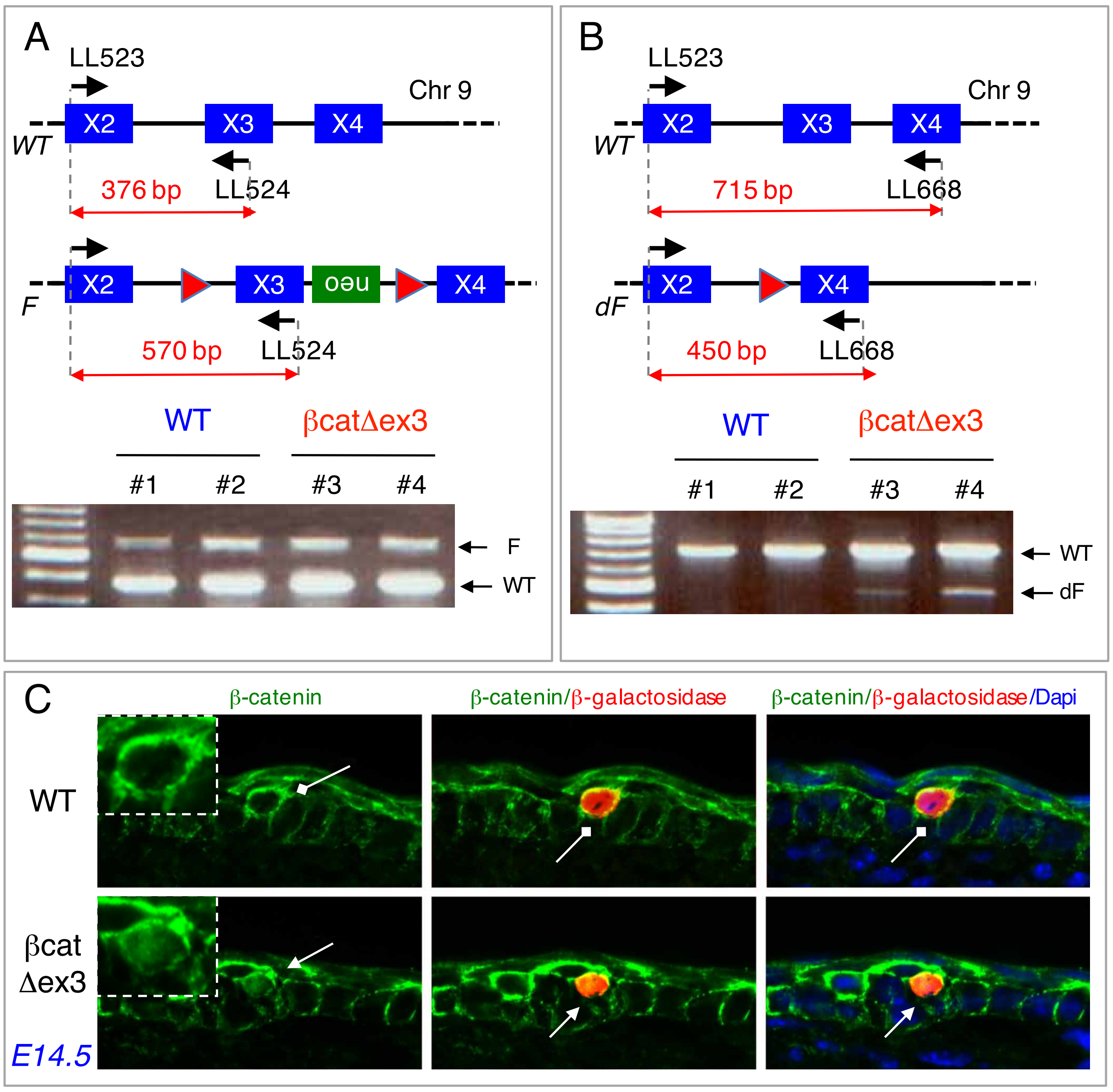


**Fig. S3. β-catenin is defloxed after Cre recombination and localized in the nucleus of mutant melanoblasts and melanocytes.**
(A,B) Schematic representation of the WT, floxed (F) and defloxed (dF) β-catenin locus. β-catenin is localized on chromosome (Chr) 9 in the mouse. A series of oligonucleotides (LL523, LL524, and LL668) was used to reveal the status of the β-catenin locus. (A) WT locus leads to a 376 bp band, and F locus to a 570bp band using the pair LL523 and LL524. (B) WT locus leads to a 715 bp band, and dF locus to a 450bp band using the pair LL523 and LL668. (C,D) Immunostaining with antibodies directed against β-catenin and β-galactosidase. Nuclei were stained with Dapi. β-galactosiade labels the Dct::LacZ-positive melanoblasts/melanocytes. (C) E14.5 WT and βcatΔex3 embryo sections. WT = (°/°; βcatex3flox/+; Dct::LacZ/°); βcatΔex3 = (Tyr::Cre/°; βcatex3flox/+; Dct::LacZ/°).

Development • Supplementary information

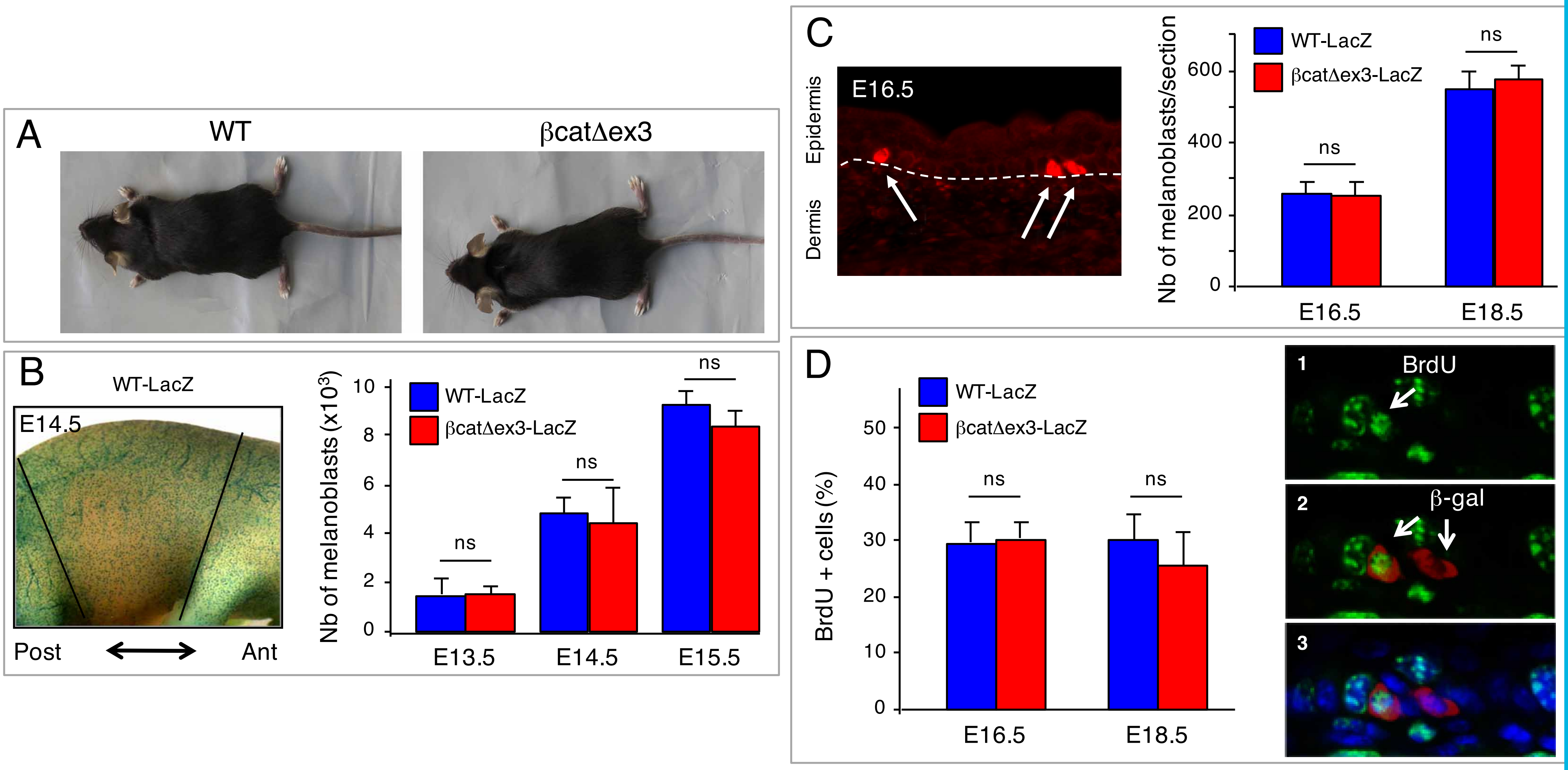


**Fig. S4. The melanocyte lineage of the dorso-lateral wave is not perturbed in Tyr::Cre/° ; βcatex3flox/+ mice.**
(A) Adult βcatΔex3 mice have the same coat/tail/ear color as control C57BL/6 mice. (B,C) β-catenin overexpression does not increase the number of truncal melanoblasts during embryonic development. (B) Macroscopic observations of WT-LacZ and βcatΔex3-LacZ embryos at E14.5 with the melanoblasts stained with X-gal for β-galactosidase activity. At E13.5, E14.5 and E15.5, the number of melanoblasts was estimated on the right side of the trunk region located between the forelimbs and hindlimbs (somites 13-25, limits shown as black lines the picture). (C) At E16.5 and E18.5, melanoblasts (red on photomicrographs and highlighted with white arrows) were counted on WT-LacZ and βcatΔex3-LacZ embryo sections immunostained for β-galactosidase. The numbers represent the melanoblasts located in the epidermis and hair follicles. (D) Determination of proliferation rate for β-galactosidase-positive cells at E16.5 and E18.5 in WT-LacZ and βcatΔex3-LacZ embryos. Between 15 and 54 sections, derived from two to four embryos from independent litters, were analyzed for each embryonic stage and each genotype. The percentages were obtained from melanoblasts of the epidermis and hair follicles. Immunofluorscence photomicrographs of a typical section show BrdU-positive cells in green (1), β-galactosidase-positive cells in red (2), and nuclei stained with Dapi in blue (3). Statistical significance was calculated with the Mann-Whitney U test and is indicated: ns, non significant. Post = posterior. Ant = anterior. WT = (°/°; βcatex3flox/+) or (Tyr::Cre; βcatex3+/+); βcatΔex3 = (Tyr::Cre/°; βcatex3flox/+); WT-LacZ = (°/°; βcatΔex3flox/+; Dct::LacZ/°) ; βcatΔex3-LacZ = (Tyr::Cre/°; βcatex3flox/+; Dct::LacZ/°).

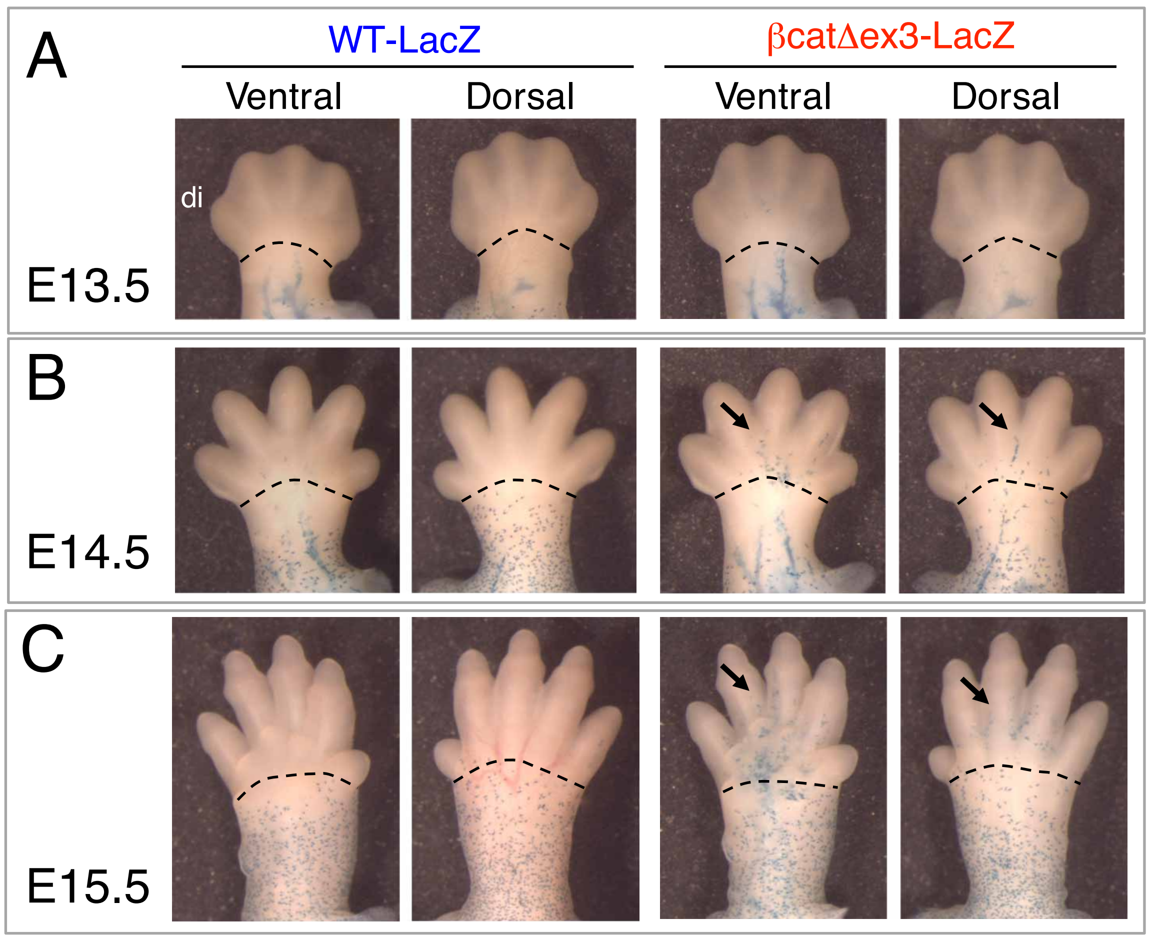


**Fig. S5. Mutant melanoblasts are present in the distal region of the paws from E14.5.** WT-LacZ and βcatΔex3-LacZ embryonic paws were stained with X-gal. At E13.5 (A), no melanoblast is observed in either WT or mutant distal (di) region of the paws. At E14.5 (B) and E15.5 (C) βcatΔex3-LacZ melanoblasts are found in the distal part of the of the anterior paws. No melanoblast is observed in WT (B,C). Dashed lines separate the distal (di) part of the limb (paw) from the proximal limb. Arrows indicate the presence of melanoblasts. WT-LacZ = (°/°; βcatex3flox/+; Dct::LacZ/°); βcatΔex3-LacZ = (Tyr::Cre/°; βcatex3flox/+; Dct::LacZ/°).

Development • Supplementary information

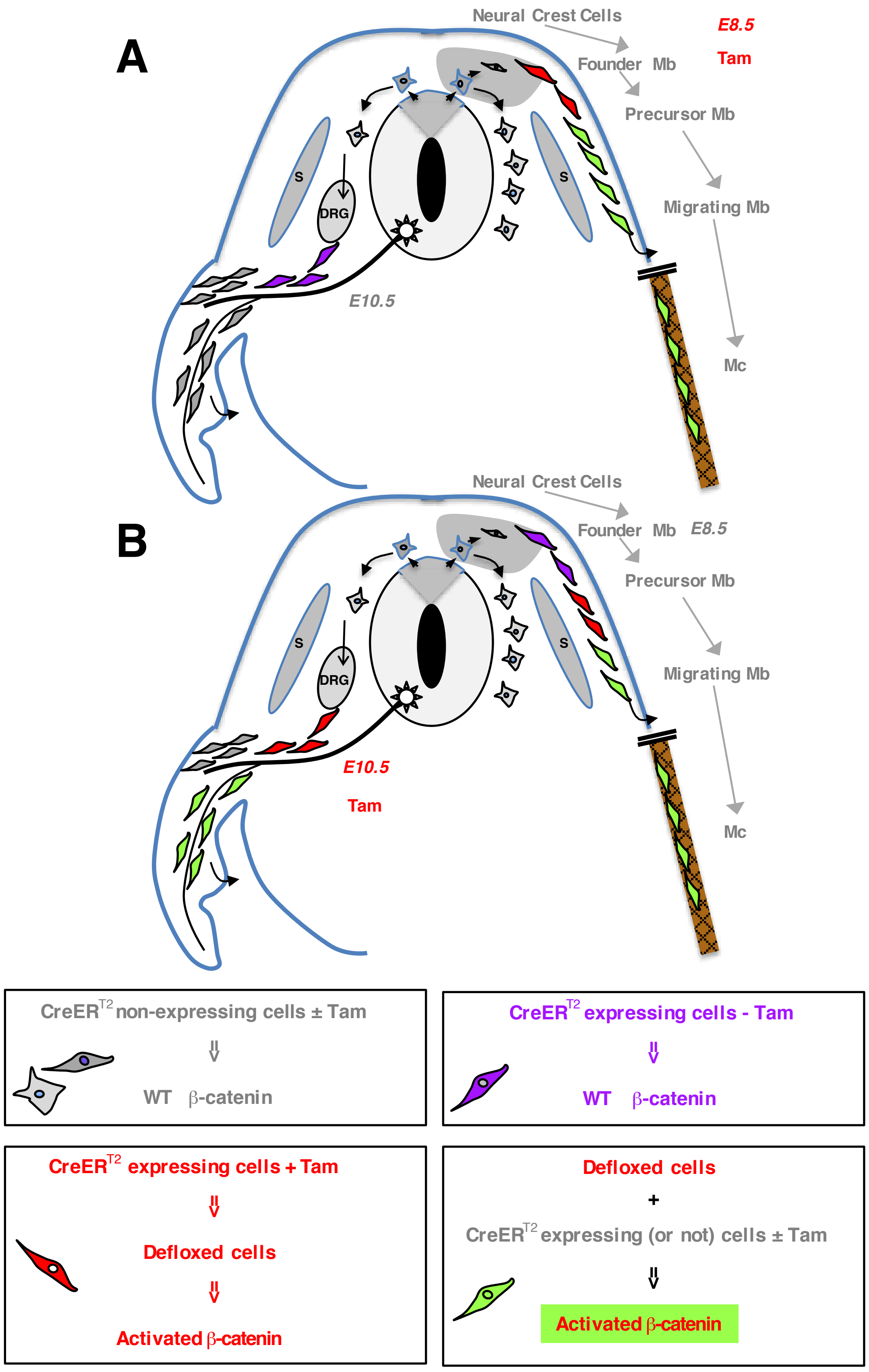


**Fig. S6. Activation of β-catenin in the first and/or second waves of melanocytes**
The Cre-ERT2 recombinase mRNA and protein are present in cells in which the tyrosinase promoter is active. Cre-ERT2 protein is active in the presence of 4OH-tamoxifen, allowing this chimeric protein to be translocated to the nucleus and to deflox in an irreversible way the floxed gene. In consequence, once the gene is defloxed, all the descendent cells will remain defloxed. The temporal activation of Cre-ERT2 is crucial for the fate of the floxed status of the melanocyte lineage. Cre-ERT2 expressing cells may induce the activation of β-catenin in the presence of tamoxifen (red). However, cells expressing Cre-ERT2 will not be defloxed in the absence of tamoxifen (purple). Cells that have been defloxed, and their descendants, are expressing an activated form of β-catenin (green). Cells that do not express Tyrosinase, and their ascendants, will never produce an activated form of β-catenin (grey). Tamoxifen was injected in pregnant mother when the embryos were E8.5 (A) or E10.5 days old (B). Tamoxifen induction occurring at E8.5 allows the activation of β-catenin in the first wave, but not in the second wave of melanocytes. At E10.5, Tamoxifen induction occurs at the time of the specification of SCP. In wild-type conditions, they are normally specified in Schwann cell but occasionally may generate some melanocytes.

Development • Supplementary information
