# Supplementary material for: Stabilization of β-catenin promotes melanocyte specification at the expense of the Schwann cell lineage
Source: Development. 2022 Jan 24;149(2):dev194407. doi: 10.1242/dev.194407 (PMC8917410; doi:10.1242/dev.194407)
Supplement: Reviewer comments [file develop-149-194407_review_history.pdf]

Stabilization of ß-catenin promotes melanocyte specification at the expense of the Schwann cell lineage

Sophie Colombo, Valérie Petit, Roselyne Y. Wagner, Delphine Champeval, Ichiro Yajima, Franck Gesbert, Zackie Aktary, Irwin Davidson, Véronique Delmas and Lionel Larue
DOI: 10.1242/dev.194407

Editor: James Briscoe

Review timeline

Original submission:	29 June 2020
Editorial decision:	31 January 2021
First revision received:	15 October 2021
Editorial decision:	23 November 2021
Second revision received:	25 November 2021
Accepted:	25 November 2021

Original submission

First decision letter

MS ID#: DEVELOP/2020/194407

MS TITLE: Stabilization of ß-catenin promotes melanocyte specification at the expense of the Schwann cell lineage

AUTHORS: Sophie Colombo, Valerie Petit, Roselyne Y Wagner, Delphine Champeval, Ichiro Yajima, Franck Gesbert, Irwin Davidson, Veronique Delmas, Lionel Larue, and Zackie Aktary

I have now received all the referees reports on the above manuscript, and have reached a decision. The referees' comments are appended below, or you can access them online: please go to BenchPress and click on the 'Manuscripts with Decisions' queue in the Author Area.

The overall evaluation is positive and we would like to publish a revised manuscript in Development. Please attend to the minor issues listed by Referee 3 and then I will be happy to accept your study.

We are aware that you may currently be unable to access the lab to undertake experimental revisions. If it would be helpful, we encourage you to contact us to discuss your revision in greater detail. Please send us a point-by-point response indicating where you are able to address concerns raised (either experimentally or by changes to the text) and where you will not be able to do so within the normal timeframe of a revision. We will then provide further guidance. Please also note that we are happy to extend revision timeframes as necessary.

Reviewer 1

Advance summary and potential significance to field

This is an absolutely wonderful paper. It focuses on elucidating key signals biasing the production of melanocytes in distant body regions represented by paws. The authors conclude that canonical Wnt

© 2022. Published by The Company of Biologists under the terms of the Creative Commons Attribution License (https://creativecommons.org/licenses/by/4.0/).

signaling acts within the nerve to shift the balance of future fates derived from nerve-associated Schwann cell precursors and going towards pigment cells or mature Schwann cells.

Comments for the author

Although the experiments are solid, and the interpretation of the data is optimal and correct, there are some minor caveats that must be finished for bridging the logic of this manuscript:
1. The authors use Tyr-Cre and Tyr-CreERT2 mouse lines. Tyr is not expected to be expressed in Schwann cell precursors or immature Schwann cells, except for very rare cases. Thus, either the current knowledge about the absence of Tyr expression in the nerves in incomplete, or the interpretation that the authors make is not entirely correct. Here I see several opportunities: Tyr-Cre lines are not adequately and naturally reproducing the tightly controlled expression of Tyr in pigment cells and show the expression of Cre also in Schwann cell lineage (being an artifact (much more leaky control of the specificity of the expression) of these Cre lines). This is alright, and makes this tool still valid. Still, this requires a check and a concrete explanation. I suggest to perform RNAscope for Tyr and Cre transcripts on top of DCT-LacZ analysis in the nerves/melanocytes. Also, please discuss it.
There might be an alternative situation as well: the expression of Tyr is activated transiently in the Schwann cell precursors, when they did not fully commit to melanocyte fate yet. Tyr, in principle, might be present for a short while in nerve-associated unstable progenitors. Many of these transient Mitf/Tyr positive cells will revert back to Schwann cell phenotype if they do not receive the stabilizing canonical Wnt signaling support. If they do receive the stabilizing support, they will fully convert to melanocyte fate and leave the nerve. If this is true, it must be shown clearly by visualizing where the natural expression of Tyr is starting, and if it can be systematically observed within the peripheral nerves and SCPs. At this point I rather think that the Cre lines leak unnaturally in SCPs, which is ok for the author's story, but should be clearly stated and supported by the data.
Alternatively, the authors can involve Sox10-CreERT2 mouse models.
2. In the abstract, the author write that the second wave of melanocytes derived from SCPs is rather small: “The first wave of melanocytes to colonize the skin is directly derived from neural crest cells, while a small number of second wave melanocytes is derived from Schwann-cell precursors (SCPs).” I do not think this is true, as it was revealed previously that the contribution of SCP-derived melanocytes is large (for instance, in chick experiments as shown by Adameyko and Kalcheim groups or around cranial nerves IX-X in mouse – Adameyko et al. Development 2012).
3. The absence of difference in melanocyte numbers in the trunk (not paws)
can be explained by the strong presence of Wnt signaling in the trunk whereas the paws can experience a deficit of canonical Wnt signaling. Thus experimental stabilization of b-cat in paw-localizing SCPs can cause their over-conversion into melanocytes, whereas it will not cause over-conversion in the trunk, as it reached its maximum already.
4. It is important to show the sections through the large and small nerves in the paw with staining (RNA scope?) for Tyr, Dct, Mitf, Sox10, Erbb3, Ngfr.
This should show where exactly the induction of new melanocytes is going on in Tyr-Cre/b-cat-DEL-ex3 or wt models.

Reviewer 2

Advance summary and potential significance to field

This manuscript adds to the evidence that there is a "second wave" of melanoblast differentiation, and perhaps puts a limit on the extent of these cells to overall pigmentation.

Comments for the author

This manuscript analyses the result of activation of beta-catenin signalling in the melanoblast lineage, using a beta-catenin gene in which exon 3 is flanked by loxP sites crossed with Cre driven by the tyosinase promoter.
Removal of exon 3 results in a constitutively active beta-catenin. The in vivo studies on mice and embryos are very well done. The work in cell culture supports their model, but by its nature is less compelling.

© 2022. Published by The Company of Biologists under the terms of the Creative Commons Attribution License (https://creativecommons.org/licenses/by/4.0/).

The abstract overstates their findings. The last sentence "In addition hyperactivation of the Wnt/ß-catenin pathway repressed FoxD3 expression which is necessary for Schwann cell development, through Mitf-M activation"
does not make it clear that the repression of FOXD3 by activating the Wnt pathway was in schwanoma cells, and they do not show any evidence that this was mediated through MITF. They should rewrite the Abstract to indicate what was done in vivo and what were cultured cell experiments.
There is thought to be more than one wave of melanoblast/melanocyte differentiation. Most derive directly from multipotent neural crest cells, a second comes from Schwann cells precursors (SCPs). It is debated how much the latter pathway contributes to overall pigmentation. The "mesodermal wave" melanoblasts referred to in the Introduction, and postulated by Kinsler and Larue (2018) are somewhat more controversial. Certainly Schwann cells and melanoblasts share a common progenitor and a certain plasticity in differentiation, and transcription factors determining either lineage may be coexpressed in these progenitor cells. Furthermore, Wnt signalling is important in several aspects of neural crest differentiation, mediated via beta-catenin. MITF is the key factor determining the melanoblast lineage and there is evidence that beta-catenin has a role in activating MITF.
In this manuscript, using the well-established Tyr:Cre lines they conditionally express the constitutively active form of beta-catenin in the melanoblast lineage(s). There is data suggesting that this transgenic Cre is active very early in the lineage, possibly in a common progenitor of melanocytes and glia.
Mice born from this cross had pigmented soles of their paws. Histology shows this is due to dermal not epidermal melanocytes. There does not appear to be any other pigmentatary change. Melanoblasts were visualised in embryos with the widely used Dct:LacZ transgene and the authors see no difference in trunk melanoblasts. However, they did see melanoblasts in beta-catenin activated mutant paws, not seen in WT. They suggest these are nerve associated (which would be consistent with a SCP origin) but they do not carry out any labelling to confirm. It would be informative if they showed sections through these embryonic limbs to indicate localization of the melanoblasts.
They go on to use a tamaxifen inducible Tyr:Cre. The ectopic melanoblasts are not seen if embryos are treated at E8.5 or E11.5, but are seen if treated at E10.5. This is curious at first sight; why does treatment at E8.5 to activate beta-catenin not produce these ectopic melanoblasts but activation later does? It must be that at E8.5 the Tyr:Cre transgene is not active in those cells that give rise to the ectopic melanoblasts, so are later developing, i.e. the later wave. They suggest at this point "that SCPs actually specify into melanocytes as early as E10.5". This is not necessarily quite true, the could be bi-potent at that stage (as they say later in the paragraph)
A key piece of data is their analysis of E15.5 paws in which they quantify a decrease in GFAP+ cells and an increase in MITF+ cells, so documenting a shift from Schwann cells to melanocytes. However, the decrease in GFAP+ is fairly small, and is smaller than the increase in MITF+. The number of independent measurements is not given in the Figure legend nor in the Methods. This is important to give confidence to the conclusion.
The final results section is titled "beta-catenin promotes the SCP-derived melanocyte fate through Mitf repression of FoxD3". This is additional work in cultured cells and the title exagerates the relevance of the cell culture work to the in vivo situation. The title should be modified.
In schwanoma cells they knock down APC using siRNA in order to activate beta catenin signalling and they note a decrease in FOXD3 expresion (which is required for Schwann cell differentation). In a converse experiment in melanoma cells, they knock down MITF, and FOXD3 goes up. The former observation is in line with their in vivo observations. The latter is suggesting that MITF acts in melanoma cells at least to repress FOXD3 expression.
To support this they look at available ChIP-seq data from human primary melanocytes and melanoma. They note an MITF binding peak in both cells at
~60kb distal from the FOXD3 gene, as well as one in melanocytes at the promoter. The data is fairly noisy, and there are several other peaks not commented on. The -60 peak in melanoma does, however, coincide with a binding peak of SOX10 (although the promoter peak does not). There is no data for SOX10 in melanocytes. There is suprisingly good sequence conservation between mouse and human genomes at this location.
They also have data for H2AZ and BRG binding in melanoma and H3K27Ac in melanocytes. There are peaks close to but not precisely coincident with the MITF -60kb peak on melanoma cells. It is not clear to me how to interpret this: they suggest MITF is repressing FOXD3 in these cells, but BRG1 and H3K27ac are associated with active enhancers. Can they comment on this.

© 2022. Published by The Company of Biologists under the terms of the Creative Commons Attribution License (https://creativecommons.org/licenses/by/4.0/).

In the Discussion they should compare and contrast their conclusions with other mutants which give very similar paw pigmentation phenotypes. The Dsk mutants characterised by van Raamsdonk et al, which are hypomorphs of Gnaq and Gna11, result in demal melanocytes in paws, as they see. Mutations in two ribosomal proteins, Rps19 and Rps20 reported by McGowan et al result in dark footpads.
Also in the Discussion they bring up acral melanoma and the possibility that this comes from second wave melanoblasts. This is only a possibility; second wave melanocytes have not been demonstrated in humans, and what contribution they may make to acral melanoma is speculative. They cannot definitively say
"while ALM arises from melanocytes derived from the second wave of melanoblasts". This should be noted as speculation.

Reviewer 3

Advance summary and potential significance to field

In this study, Colombo et al. have performed elegant lineage tracing studies using a transgenic mouse model that allows inducible activation of ss-catenin under the tyrosine kinase promoter (Tyr-Cre:ßcatex3flox/+). By activating b-catenin at various stages during development, the authors suggest that activation of b-catenin in SCPs favours lineage specification to melanocytes instead of Schwann cells in part of the paws, but not in the trunk region. Mechanistically, they showed that expression of b-catenin induces the transcription factor MITF which is critical for melanocyte differentiation. Concomitantly, the expression of the transcription factor FoxD3, which is instrumental for Schwann cell lineage specification, is repressed.

This is a potentially interesting and comprehensive study that deserves publication in this journal, provided that the issues below can be addressed.

Comments for the author

Main points:
1) The Tyr::CreA driver used in this study was originally described by the same authors as being highly specific for the melanocyte lineage (Delmas et al. 2003). For Tyr::CreA-driven and Tyr::CreERT2-driven ß-catenin overexpression to promote a fate switch in SCPs, the driver has obviously to be expressed in SCPs.
This needs to be shown, in particular around the time (E10.5) when Tyr::CreERT2 induction leads to the observed phenotype. Showing increased nuclear ß-catenin in SCPs, anti-Cre labeling, and/or the use of a recombination tracer would help addressing this point.
2) Along these lines, is Cre in Tyr::CreA and Tyr::CreERT2, respectively, predominantly expressed in SCPs of limbs, which could explain the region specificity of the observed phenotype?
3) An alternative explanation for the observed phenotype is activation of a melanoblast fate in migratory neural crest cells, in analogy to Hari et al, Development 2012, who showed massive induction and expansion of melanoblasts at ectopic sites upon ß-catenin activation in neural crest cells around E9.5. How can the authors exclude this possibility in their system? I suggest that the authors at least discuss this possibility.
Are, in their experiments, melanocytes present at ectopic sites other than the palms, e.g. in sympathetic ganglia, spleen, etc., as seen by Hari et al., 2012?
4) No phenotype is observed upon induction of Tyr::CreERT2 at E8.5., but is Tyr::CreERT2 already expressed at this early stage?
5) In Figure 4, the GFAP staining seems to be nuclear, although it is supposed to be cytoplasmic.
6) In Figure 5, it would be preferable to use a non-pathogenic Schwann cell line for the in vitro experiments shown here.
7) In Figure 6, the schematic is shown but there is no figure legend for this figure, this should be added.
8) Although this might be beyond the scope of the present paper, it would be interesting to address whether inactivation of ß-catenin signaling in SCPs in vivo would result in fewer melanocytes and more Schwann cells.

© 2022. Published by The Company of Biologists under the terms of the Creative Commons Attribution License (https://creativecommons.org/licenses/by/4.0/).

Minor point:
1) In Figure S4B, the genotype of the mouse in the representative image should be labelled.

First revision

Author response to reviewers' comments

Reviewer 1 Advance Summary and Potential Significance to Field:
This is an absolutely wonderful paper. It focuses on elucidating key signals biasing the production of melanocytes in distant body regions represented by paws. The authors conclude that canonical Wnt signaling acts within the nerve to shift the balance of future fates derived from nerve-associated Schwann cell precursors and going towards pigment cells or mature Schwann cells.

* We thank the reviewer for this positive comment.

Reviewer 1 Comments for the Author:
Although the experiments are solid, and the interpretation of the data is optimal and correct, there are some minor caveats that must be finished for bridging the logic of this manuscript:

1Q1. The authors use Tyr-Cre and Tyr-CreERT2 mouse lines. Tyr is not expected to be expressed in Schwann cell precursors or immature Schwann cells, except for very rare cases. Thus, either the current knowledge about the absence of Tyr expression in the nerves in incomplete, or the interpretation that the authors make is not entirely correct. Here I see several opportunities: Tyr-Cre lines are not adequately and naturally reproducing the tightly controlled expression of Tyr in pigment cells and show the expression of Cre also in Schwann cell lineage (being an artifact (much more leaky control of the specificity of the expression) of these Cre lines). This is alright, and makes this tool still valid. Still, this requires a check and a concrete explanation. Also, please discuss it.
There might be an alternative situation as well: the expression of Tyr is activated transiently in the Schwann cell precursors, when they did not fully commit to melanocyte fate yet. Tyr, in principle, might be present for a short while in nerve-associated unstable progenitors. Many of these transient Mitf/Tyr positive cells will revert back to Schwann cell phenotype if they do not receive the stabilizing canonical Wnt signaling support. If they do receive the stabilizing support, they will fully convert to melanocyte fate and leave the nerve. If this is true, it must be shown clearly by visualizing where the natural expression of Tyr is starting, and if it can be systematically observed within the peripheral nerves and SCPs. At this point, I rather think that the Cre lines leak unnaturally in SCPs, which is ok for the author's story, but should be clearly stated and supported by the data.
Alternatively, the authors can involve Sox10-CreERT2 mouse models.

1A1. As the referee mentions “Tyr is not expected to be expressed in Schwann cell precursors or immature Schwann cells, except for very rare cases.” We agree with this statement. Indeed, it has been shown that Schwann cells and melanocytes are very close for several reasons besides the fact that they are derived from the neural crest. It has been shown that human melanocytes can transdifferentiate in Schwann cells (Chi et al.). Spindle cell melanoma and pigmented neurofibroma possess cells with melanocyte and Schwann cell characteristics, with cells producing weakly Mitf and Tyrosinase. It gives to these cells the potential opportunity to perform a bidirectional differentiation (Motoi et al., 2005; Winnepenninckx et al., 2003). Melanotic schwannoma, a rare variant of nerve sheath tumors that arise from spinal nerve roots, is composed of neoplastic Schwann cells that produce melanin (Alexiev et al., 2018). However, the molecular status of these cells were not described but we may hypothesize that they produce Tyrosinase and therefore Mitf. Moreover, Tyrosinase promoter activity was detected in other cells than melanocytes such as the cortex, olfactory system, hippocampus, epithalamus, and substantia nigra during embryonic development (Tief et al., 1998). Finally, adult melanocytes, expressing Tyrosinase, were present in many tissue/organ that was not initially suspected (see for instance review, (Brito and Kos, 2008; Colombo et al., 2011; Gudjohnsen et al., 2015; Yajima and Larue, 2008)). It has already been shown that the use of the Tyr::Cre transgene, to conditionally delete specific genes, is targeting

© 2022. Published by The Company of Biologists under the terms of the Creative Commons Attribution License (https://creativecommons.org/licenses/by/4.0/).

the melanocytic lineage but also the enteric nervous system and the Schwann cell lineage (Puig et al., 2009; Radu et al., 2019). We added this information in the introduction and discussion.

In order to answer directly the referee, we followed the expression of GFP (corresponding to the cells that were defloxed by Cre) and GFAP (used as a marker for Schwann cells and Schwann cell precursors) in Tyr::Cre/° ; ZEG/° E14.5 embryo. We observed that cells are both GFP and GFAP positive in the ventral part of the limbs. This double labelling revealed that GFAP positive cells expressed or are derived from a cell that produced Cre under the control of the tyrosinase promoter. This information is given in the text as a new Figure (see Figure 5).

1Q2. In the abstract, the author write that the second wave of melanocytes derived from SCPs is rather small: “The first wave of melanocytes to colonize the skin is directly derived from neural crest cells, while a small number of second wave melanocytes is derived from Schwann-cell precursors (SCPs).” I do not think this is true, as it was revealed previously that the contribution of SCP-derived melanocytes is large (for instance, in chick experiments as shown by Adameyko and Kalcheim groups or around cranial nerves IX-X in mouse – Adameyko et al. Development 2012).

1A2. Since the goal of this article is not to evaluate the importance of the contribution of the first or the second wave of melanocytes, we modified the text accordingly by removing “small number” in the sentence “The first wave of melanocytes to colonize the skin is directly derived from neural crest cells, while the second wave of melanocytes is derived from Schwann-cell precursors (SCPs).”

1Q3. The absence of difference in melanocyte numbers in the trunk (not paws) can be explained by the strong presence of Wnt signaling in the trunk, whereas the paws can experience a deficit of canonical Wnt signaling. Thus, experimental stabilization of b-cat in paw-localizing SCPs can cause their over-conversion into melanocytes, whereas it will not cause over-conversion in the trunk, as it reached its maximum already.

1A3. We fully agree with the referee and we thank him/her. It is a plausible hypothesis that we discussed. Indeed, it is important to note that there was a difference in the paws but not in the trunk on this genetic background. Such difference could be due to differential regulation of the endogenous Wnt signalling pathway in these two different environments.

1Q4. It is important to show the sections through the large and small nerves in the paw with staining (RNA scope?) for Tyr, Dct, Mitf, Sox10, Erbb3, Ngfr. This should show where exactly the induction of new melanocytes is going on in Tyr-Cre/b-cat-DEL-ex3 or wt models.

1A4.
It is indeed an important question but unfortunately, we are not able to properly answer this question. To show this, we should perform a movie of the labelling of the markers of interest in WT and mutant embryos from E10.5 to E14.5. However, we followed the expression of GFP (corresponding to the cells that were defloxed by Cre) and Tujj1 (used as a marker for neuronal cells) in Tyr::Cre/° ; ZEG/° E14.5 embryo (Figure 3A-C). We observed that some cells are close from one to another being GFP or Tujj1 positive in the ventral part of the limbs. This double labelling revealed that defloxed cells (melanoblast or Schwann cells) are closed to neurons. This result is shown in Figure 3. This experiment was indeed not sufficient to show that melanoblasts were interacting with these neurons. In this respect, we performed the staining of Pmel and Tuj1 in paws transversal sections revealing that some Pmel-positive cells are in close proximities with Tuj1-positive cells (Figure 3D-K). We tried to perform triple labelling directed against GFP, Pmel (a melanocyte marker) and Tujj1, but unfortunately, we were unsuccessful after using different associations of commercial antibodies.

Reviewer 2 Advance Summary and Potential Significance to Field:

This manuscript adds to the evidence that there is a "second wave" of melanoblast differentiation, and perhaps puts a limit on the extent of these cells to overall pigmentation.

Reviewer 2 Comments for the Author:
This manuscript analyses the result of activation of beta-catenin signalling in the melanoblast lineage, using a beta-catenin gene in which exon 3 is flanked by loxP sites crossed with Cre driven

© 2022. Published by The Company of Biologists under the terms of the Creative Commons Attribution License (https://creativecommons.org/licenses/by/4.0/).

by the tyosinase promoter. Removal of exon 3 results in a constitutively active beta-catenin. The in vivo studies on mice and embryos are very well done. The work in cell culture supports their model, but by its nature is less compelling.

* We thank the reviewer for this positive comment on the *in vivo* studies. We strengthen the *in vitro* part to better support our conclusions.

2Q1. The abstract overstates their findings. The last sentence "In addition, hyperactivation of the Wnt/ß-catenin pathway repressed FoxD3 expression, which is necessary for Schwann cell development, through Mitf-M activation" does not make it clear that the repression of FOXD3 by activating the Wnt pathway was in schwanoma cells, and they do not show any evidence that this was mediated through MITF. They should rewrite the Abstract to indicate what was done in vivo and what were cultured cell experiments.

2A1. It was indeed a shortcut due to the size constraints of the abstract. However, the main text was not ambiguous. Additionally, to human schwannoma cells, we performed some *in vitro* experiments in non-transformed human Schwann cells and could show that the activation of the Wnt/¤-catenin pathway was able to repress FOXD3 using siAPC (Figure 7). We modified the sentence in the abstract to “In addition, hyperactivation *in vitro* of the Wnt/ß-catenin pathway repressed FoxD3 expression in Schwann cells, which would be necessary for melanocyte development.”

2Q2. There is thought to be more than one wave of melanoblast/melanocyte differentiation. Most derive directly from multipotent neural crest cells, a second comes from Schwann cells precursors (SCPs). It is debated how much the latter pathway contributes to overall pigmentation. The "mesodermal wave" melanoblasts referred to in the Introduction, and postulated by Kinsler and Larue (2018) are somewhat more controversial. Certainly Schwann cells and melanoblasts share a common progenitor and a certain plasticity in differentiation, and transcription factors determining either lineage may be coexpressed in these progenitor cells. Furthermore, Wnt signalling is important in several aspects of neural crest differentiation, mediated via beta-catenin. MITF is the key factor determining the melanoblast lineage, and there is evidence that beta-catenin has a role in activating MITF.

In this manuscript, using the well-established Tyr:Cre lines they conditionally express the constitutively active form of beta-catenin in the melanoblast lineage(s). There is data suggesting that this transgenic Cre is active very early in the lineage, possibly in a common progenitor of melanocytes and glia. Mice born from this cross had pigmented soles of their paws. Histology shows this is due to dermal not epidermal melanocytes. There does not appear to be any other pigmentatary change. Melanoblasts were visualised in embryos with the widely used Dct:LacZ transgene and the authors see no difference in trunk melanoblasts. However, they did see melanoblasts in beta-catenin activated mutant paws, not seen in WT. They suggest these are nerve associated (which would be consistent with a SCP origin) but they do not carry out any labelling to confirm. It would be informative if they showed sections through these embryonic limbs to indicate localization of the melanoblasts.

2A2. We performed the staining of Pmel and Tuj1 in paws transversal sections revealing that some Pmel-positive cells are in close proximities with Tuj1-positive cells (Figure 3D-K). We tried to perform a triple labelling directed against GFP, Pmel (a melanocyte marker) and Tujj1, but unfortunately we were unsuccessful after using different association of commercial antibodies.

2Q3. They go on to use a tamaxifen inducible Tyr:Cre. The ectopic melanoblasts are not seen if embryos are treated at E8.5 or E11.5, but are seen if treated at E10.5. This is curious at first sight; why does treatment at E8.5 to activate beta-catenin not produce these ectopic melanoblasts but activation later does? It must be that at E8.5 the Tyr:Cre transgene is not active in those cells that give rise to the ectopic melanoblasts, so are later developing, i.e. the later wave. They suggest at this point "that SCPs actually specify into melanocytes as early as E10.5". This is not necessarily quite true, the could be bi-potent at that stage (as they say later in the paragraph).

2A3. We modified the text by writing “that bipotent SCPs may specify into melanocytes as early as E10.5 when the level of bcat is higher than normal.”

© 2022. Published by The Company of Biologists under the terms of the Creative Commons Attribution License (https://creativecommons.org/licenses/by/4.0/).

2Q4. A key piece of data is their analysis of E15.5 paws in which they quantify a decrease in GFAP+ cells and an increase in MITF+ cells, so documenting a shift from Schwann cells to melanocytes. However, the decrease in GFAP+ is fairly small, and is smaller than the increase in MITF+. The number of independent measurements is not given in the Figure legend nor in the Methods. This is important to give confidence to the conclusion.

2A4. We are sorry that we did not include the number of independent measurements in Figure 6, the total number of WT and mutant embryos is equal to 8, four for each genotype. This is now added in the figure legend.

2Q5. The final results section is titled "beta-catenin promotes the SCP-derived melanocyte fate through Mitf repression of FoxD3". This is additional work in cultured cells and the title exagerates the relevance of the cell culture work to the in vivo situation. The title should be modified.

2A5. We modified the title of this result section to “□-catenin induces the transcription of Mitf and the repression of FoxD3”.

2Q6. In schwanoma cells they knock down APC using siRNA in order to activate beta catenin signalling and they note a decrease in FOXD3 expression (which is required for Schwann cell differentiation). In a converse experiment in melanoma cells, they knock down MITF, and FOXD3 goes up. The former observation is in line with their in vivo observations. The latter is suggesting that MITF acts in melanoma cells at least to repress FOXD3 expression.

To support this they look at available ChIP-seq data from human primary melanocytes and melanoma. They note an MITF binding peak in both cells at ~60kb distal from the FOXD3 gene, as well as one in melanocytes at the promoter. The data is fairly noisy, and there are several other peaks not commented on. The -60 peak in melanoma does, however, coincide with a binding peak of SOX10 (although the promoter peak does not). There is no data for SOX10 in melanocytes. There is suprisingly good sequence conservation between mouse and human genomes at this location.

2A6. The referee is correct, there are several other peaks at the putative FOXD3 enhancer. To illustrate our reply, we include here a close-up view of this enhancer region. There are at least 4 MITF bound sites in 501Mel cells that are evident in this region (labelled regions 1-4 in the figure). We focused on site 4 for several reasons. The MITF site is occupied in melanocytes, it is adjacent to a SOX10 bound site, it is associated with strong occupancy of BRG1 and H2AZ and marking by H3K27ac. All of these are hallmarks of active enhancer elements of melanoma cells. The requirement for SOX10 as a mark of the most functionally relevant sites was previously described in (Laurette et al., 2015) and has been recently highlighted by studies from the Aerts laboratory see for instance (Wouters et al., 2020). Nevertheless, MITF site 1 is also occupied in melanocytes and is associated with H3K27ac, but shows much lower BRG1 and H2AZ occupancy. Sites 2 and 3 are not occupied in melanocytes and but show weak or no H3K27ac. We also note that regions 5-7 have all of the characteristics of regulatory elements with clear nucleosome depleted regions. These are very likely binding sites for transcription factors other than MITF and SOX10 studied here. We have revised the text to include a discussion of the other sites and we modified the panel I of figure 7.

© 2022. Published by The Company of Biologists under the terms of the Creative Commons Attribution License (https://creativecommons.org/licenses/by/4.0/).

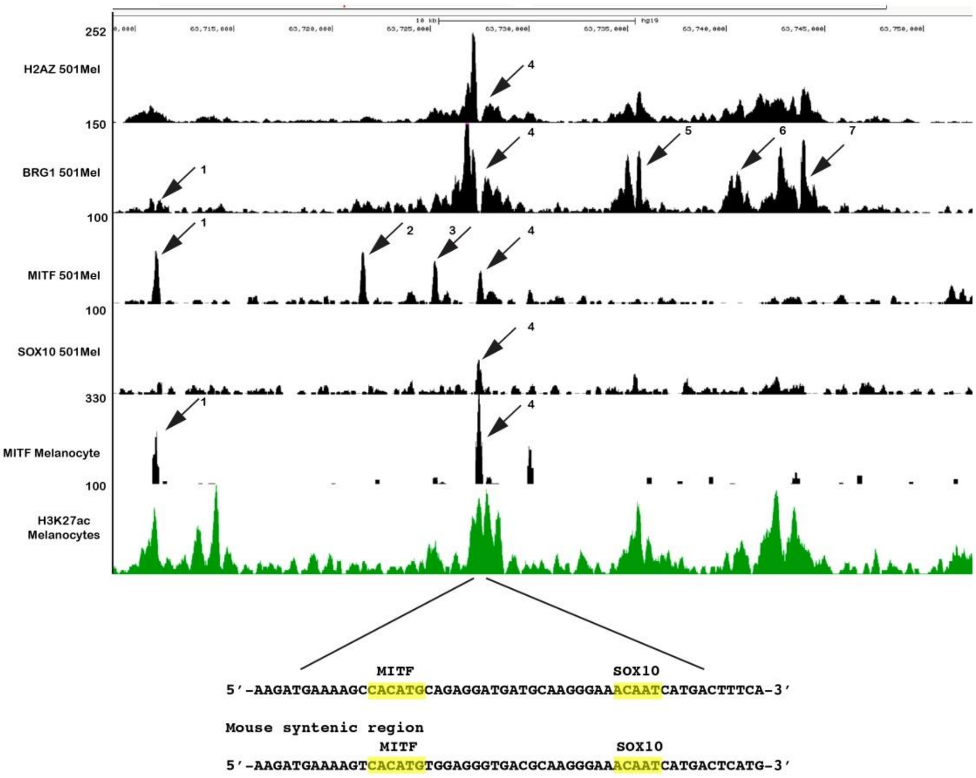

2Q7. They also have data for H2AZ and BRG binding in melanoma and H3K27Ac in melanocytes. There are peaks close to but not precisely coincident with the MITF -60kb peak on melanoma cells. It is not clear to me how to interpret this: they suggest MITF is repressing FOXD3 in these cells, but BRG1 and H3K27ac are associated with active enhancers. Can they comment on this.

2A7. The referee raises an interesting issue. It is indeed well accepted that H3K27ac is a mark of active enhancer elements. BRG1 on the other hand can also be associated with negative regulation. On this basis, at first sight, it would be logical to assign the -60kb region as an enhancer. Nevertheless, we have in the past noted (Laurette et al., 2015), numerous examples of genes whose flanking regions comprise MITF sites with the characteristics of enhancers, yet the expression of these genes is up-regulated when MITF is repressed. Whether and how MITF is acting as a repressor at these regions remains to be defined. In the case of the FoxD3 locus, it is also possible that some of the other MITF bound sites may be implicated in repression accounting for the increase in expression upon MITF silencing. Addressing these issues in a comprehensive manner will require extensive genome editing and/or use of artificial reporter assays and is obviously beyond the scope of this paper. We have modified the discussion to take these points into account.

2Q8. In the Discussion they should compare and contrast their conclusions with other mutants which give very similar paw pigmentation phenotypes. The Dsk mutants characterised by van Raamsdonk et al, which are hypomorphs of Gnaq and Gna11, result in demal melanocytes in paws, as they see. Mutations in two ribosomal proteins, Rps19 and Rps20 reported by McGowan et al result in dark footpads.

2A8. In the discussion, we already mentioned some mutants. We added other examples in the discussion as the reviewer suggested. Other mutations induce hyperpigmentation of the paws. Mutations in Gnaq (Dsk1 – V179M, and Dsk10 – F335L) and Gna11 (Dsk7 – I63V) lead to hyperpigmentation not only of the paws but also in pinna, tails of adult mice and in hair-bearing skin. Gnaq and Gna11 are the main mediators of EndrB, a key regulator of melanocyte proliferation and survival (Jain et al., 2020; Van Raamsdonk et al., 2004). Loss of function mutations in two ribosomal proteins, Rps19 (Dsk3 – T316A/Y54N) and Rps20 (Dsk4 – T29C/L32P and T2201A in 3' UTR), lead to hyperpigmentation due to an increase of melanocytes not only of the paws but also in pinna, and tails of adult mice. Interestingly, heterozygous Rps19 or Rps20 mutation in keratinocyte activate p53 that induces the level of Kitl to induce Kit present at the surface of melanocytes

© 2022. Published by The Company of Biologists under the terms of the Creative Commons Attribution License (https://creativecommons.org/licenses/by/4.0/).

(McGowan et al., 2008).

2Q9. Also in the Discussion they bring up acral melanoma and the possibility that this comes from second wave melanoblasts. This is only a possibility; second wave melanocytes have not been demonstrated in humans, and what contribution they may make to acral melanoma is speculative. They cannot definitively say "while ALM arises from melanocytes derived from the second wave of melanoblasts". This should be noted as speculation.

2A9. We thought it was clear that it was a speculation. We now clearly included this p art as a speculation. We modified the text as follow "one could speculate that ALM could arise from melanocytes derived from the second wave of melanoblasts".

Reviewer 3 Advance Summary and Potential Significance to Field:
In this study, Colombo et al. have performed elegant lineage tracing studies using a transgenic mouse model that allows inducible activation of ss-catenin under the tyrosine kinase promoter (Tyr-Cre:ßcatex3flox/+). By activating b-catenin at various stages during development, the authors suggest that activation of b-catenin in SCPs favours lineage specification to melanocytes instead of Schwann cells in part of the paws, but not in the trunk region. Mechanistically, they showed that expression of b-catenin induces the transcription factor MITF, which is critical for melanocyte differentiation. Concomitantly, the expression of the transcription factor FoxD3, which is instrumental for Schwann cell lineage specification, is repressed.
This is a potentially interesting and comprehensive study that deserves publication in this journal, provided that the issues below can be addressed.

* We thank the reviewer for this positive comment.

Reviewer 3 Comments for the Author:

Main points:
3Q1. The Tyr::CreA driver used in this study was originally described by the same authors as being highly specific for the melanocyte lineage (Delmas et al. 2003). For Tyr::CreA-driven and Tyr::CreERT2-driven b- catenin overexpression to promote a fate switch in SCPs, the driver has obviously to be expressed in SCPs.
This needs to be shown, in particular around the time (E10.5) when Tyr::CreERT2 induction leads to the observed phenotype. Showing increased nuclear b-catenin in SCPs, anti-Cre labeling, and/or the use of a recombination tracer would help addressing this point. Along these lines, is Cre in Tyr::CreA and Tyr::CreERT2, respectively, predominantly expressed in SCPs of limbs, which could explain the region specificity of the observed phenotype?

3A1. It has been shown that Schwann cells and melanocytes are very close for several reasons besides the fact that they are derived from the neural crest. It has been shown that human melanocytes can transdifferentiate in Schwann cells (Chi et al., 2011). Spindle cell melanoma and pigmented neurofibroma possess cells with melanocyte and Schwann cell characteristics, with cells producing weakly Mitf and Tyrosinase. It gives to these cells the potential opportunity to perform a bidirectional differentiation (Motoi et al., 2005; Winnepenninckx et al., 2003). Melanotic schwannoma, a rare variant of nerve sheath tumors that arise from spinal nerve roots, is composed of neoplastic Schwann cells that produce melanin (Alexiev et al., 2018). However, the molecular status of these cells was not described but we may hypothesize that they produce Tyrosinase and therefore Mitf. Moreover, Tyrosinase promoter activity was detected in other cells than melanocytes such as the cortex, olfactory system, hippocampus, epithalamus, and substantia nigra during embryonic development (Tief et al., 1998). Finally, adult melanocytes, expressing Tyrosinase, were present in many tissue/organ that was not initially suspected (see for instance review, (Brito and Kos, 2008; Colombo et al., 2011; Gudjohnsen et al., 2015; Yajima and Larue, 2008)). We added this information in the discussion. It has already been shown that the use of the Tyr::Cre transgene, to conditionally delete specific genes, is targeting the melanocytic lineage but also the enteric nervous system, smooth muscle cells in the heart, and the Schwann cell lineage (Puig et al., 2009; Radu et al., 2019; Yajima et al., 2013).
Moreover, we followed the expression of a molecular tracer, GFP (corresponding to the cells that were defloxed by Cre) and GFAP (used as a marker for Schwann cells and Schwann cell precursors) in Tyr::Cre/° ; ZEG/° E14.5 embryo. We observed that cells are both GFP and GFAP

© 2022. Published by The Company of Biologists under the terms of the Creative Commons Attribution License (https://creativecommons.org/licenses/by/4.0/).

positive in the ventral part of the limbs. This double labelling revealed that GFAP positive cells expressed or are derived from a cell that produced Cre under the control of the tyrosinase promoter. This information is given in the text as Figure 5.

As suggested by the referee, we tried to show the increase of nuclear □-catenin in SCP at E10.5 and E11.5 but we were not able to find such cell with □-catenin in the nucleus. In cell culture, it is very easy to observe □-catenin in the nucleus, but *in vivo* it is, unfortunately, not. As a reminder, Chazaud and Rossant looked for the presence of □-catenin in the nucleus of mesodermal cells at the time of gastrulation in mouse, this event was very rare even though it is clearly established that □-catenin is required to properly induce the third embryonic layer (Chazaud and Rossant, 2006)

3Q2. An alternative explanation for the observed phenotype is activation of a melanoblast fate in migratory neural crest cells, in analogy to Hari et al, Development 2012, who showed massive induction and expansion of melanoblasts at ectopic sites upon b-catenin activation in neural crest cells around E9.5. How can the authors exclude this possibility in their system? I suggest that the authors at least discuss this possibility.

3A2. In Hari et al., 2012, the authors express an active form of b-cat using the Sox10-Cre mice. bcat is known to induce the expression of Mitf, the master gene of the melanocyte lineage. In this respect these cells will have the potentiality to be specified as melanoblasts and Mitf will induce Tyrosinase. In our case using Tyr::Cre, the situation is different, the cells are already producing Tyrosinase and have already the potentiality to differentiate in melanoblasts. In other words, in one case the cells are not engaged towards the melanocyte lineage and the other the cells are already committed towards this lineage.

3Q3. Are, in their experiments, melanocytes present at ectopic sites other than the palms, e.g. in sympathetic ganglia, spleen, etc., as seen by Hari et al., 2012?

3A3. Using Wnt1::Cre or Tyr::Cre mice to delete the exon 3 of □-catenin, melanocytes were present at higher level than normal in various part of the body including sympathetic ganglia, spleen, heart and brain (Yajima et al., 2013; Yajima and Larue, 2008). However, using Tyr::CreERt2 with a tamoxifen induction at E10.5, we did not observe melanocytes at ectopic sites other than the palms. This information is now included.

3Q4. No phenotype is observed upon induction of Tyr::CreERT2 at E8.5., but is Tyr::CreERT2 already expressed at this early stage?

3A4. Indeed, we do not have the proof that CreERT2 is produced at E8.5. In this respect, we know there is a window of action that is closed at E11.5. However, we do not know yet when this window is formally open. We mentioned this point in the discussion.

3Q5. In Figure 4, the GFAP staining seems to be nuclear, although it is supposed to be cytoplasmic.

3A5. We thank the reviewer; it is indeed surprizing at a first glance. It is a 3D reconstruction, we accumulate the signal over and below the nucleus. We emphasize this point in the figure legend (currently Figure 6).

3Q7. In Figure 5, it would be preferable to use a non-pathogenic Schwann cell line for the in vitro experiments shown here.

3A7. We agree. We used also used a non-pathogenic Schwann cell line (IPN 02.3) to generalize these results. After reducing the level of APC, we obtained the same results as HEI-193 schwannoma cells (reduction of FOXD3 and in induction of AXIN2) - see figure 7.

3Q8. In Figure 6, the schematic is shown but there is no figure legend for this figure, this should be added.

3A8. We thank the reviewer for noticing this omission, we added a legend to figure 8.

© 2022. Published by The Company of Biologists under the terms of the Creative Commons Attribution License (https://creativecommons.org/licenses/by/4.0/).

3Q9. Although this might be beyond the scope of the present paper, it would be interesting to address whether inactivation of b-catenin signaling in SCPs in vivo would result in fewer melanocytes and more Schwann cells.

3A9. This point is indeed of clear interest. As the referee pointed out, it is beyond the scope of this article.

Minor point:
1) In Figure S4B, the genotype of the mouse in the representative image should be labelled.

It is now done.

References

Alexiev, B. A., Chou, P. M. and Jennings, L. J. (2018). Pathology of Melanotic Schwannoma. *Arch Pathol Lab Med* **142**, 1517-1523.

Brito, F. C. and Kos, L. (2008). Timeline and distribution of melanocyte precursors in the mouse heart. *Pigment cell & melanoma research* **21**, 464-470.

Chazaud, C. and Rossant, J. (2006). Disruption of early proximodistal patterning and AVE formation in Apc mutants *Development* **133**, 3379-3387.

Chi, G. F., Kim, D. W., Jiang, M. H., Yoon, K. J. and Son, Y. (2011). Schwann-like cells from human melanocytes and their fate in sciatic nerve injury. *Neuroreport* **22**, 603-608.

Colombo, S., Berlin, I., Delmas, V. and Larue, L. (2011). Classical and non-classical melanocytes in vertebrates. In *Melanins and melanosomes* (ed. P. A. Riley & J. Borovansky), pp. 21-51. Weinheim: Wiley-VCH Verlag & Co.

Dunn, K. J., Williams, B. O., Li, Y. and Pavan, W. J. (2000). Neural crest-directed gene transfer demonstrates Wnt1 role in melanocyte expansion and differentiation during mouse development. *Proc Natl Acad Sci U S A* **97**, 10050-10055.

Gudjohnsen, S. A., Atacho, D. A., Gesbert, F., Raposo, G., Hurbain, I., Larue, L., Steingrimsson, E. and Petersen, P. H. (2015). Meningeal Melanocytes in the Mouse: Distribution and Dependence on. *Front Neuroanat* **9**, 149.

Hari, L., Brault, V., Kleber, M., Lee, H. Y., Ille, F., Leimeroth, R., Paratore, C., Suter, U., Kemler, R. and Sommer, L. (2002). Lineage-specific requirements of beta-catenin in neural crest development. *J Cell Biol* **159**, 867-880.

Jain, F., Longakit, A., Huang, J. L. and Van Raamsdonk, C. D. (2020). Endothelin signaling promotes melanoma tumorigenesis driven by constitutively active GNAQ. . *Pigment Cell Melanoma Res.* **33**, 834-849.

Laurette, P., Strub, T., Koludrovic, D., Keime, C., Le Gras, S., Seberg, H., Van Otterloo, E., Imrichova, H., Siddaway, R., Aerts, S., et al. (2015). Transcription factor MITF and remodeller BRG1 define chromatin organisation at regulatory elements in melanoma cells. *Elife* **4**.

McGowan, K. A., Li, J. Z., Park, C. Y., Beaudry, V., Tabor, H. K., Sabnis, A. J., Zhang, W., Fuchs, H., Hrabe de Angelis, M., Myers, R. M., et al. (2008). Ribosomal mutations cause p53-mediated dark skin andpleiotropic effects. *Nature Genetics* **40**, 963-970.

Motoi, T., Ishida, T., Kawato, A., Motoi, N. and Fukayama, M. (2005). Pigmented neurofibroma: review of Japanese patients with an analysis of melanogenesis demonstrating coexpression of c-met protooncogene and microphthalmia-associated transcription factor. *Hum Pathol.* **36**, 871-877.

Puig, I., Champeval, D., De Santa Barbara, P., Jaubert, F., Lyonnet, S. and Larue, L. (2009). Deletion of Pten in the mouse enteric nervous system induces ganglioneuromatosis and mimics intestinal pseudoobstruction. *J Clin Invest* **119**, 3586-3596.

Radu, A. G., Torch, S., Fauvelle, F., Pernet-Gallay, K., Lucas, A., Blervaque, R., Delmas, V., Schlattner, U., Lafanechère, L., Hainaut, P., et al. (2019). LKB1 specifies neural crest cell fates through pyruvate-alanine cycling. *Sci Adv.* **5**.

Tief, K., Schmidt, A. and Beermann, F. (1998). New evidence for presence of tyrosinase in substantia nigra, forebrain and midbrain. *Brain Res Mol Brain Res* **53**, 307-310.

Van Raamsdonk, C. D., Fitch, K. R., Fuchs, H., Hrabé de Angelis, M. and Barsh, G. S. (2004). Effects of G-protein mutations on skin color. *Nat Genet.* **36**, 961–968.

Winnepenninckx, V., De Vos, R., Stas, M. and van den Oord, J. J. (2003). New phenotypical and ultrastructural findings in spindle cell (desmoplastic/neurotropic) melanoma **11**, 319-325.

© 2022. Published by The Company of Biologists under the terms of the Creative Commons Attribution License (https://creativecommons.org/licenses/by/4.0/).

Wouters, J., Kalender-Atak, Z., Minnoye, L., Spanier, K. I., De Waegeneer, M., Bravo González-Blas, C., Mauduit, D., Davie, K., Hulselmans, G., Najem, A., et al. (2020). Robust gene expression programs underlie recurrent cell states and phenotype switching in melanoma. . *Nat Cell Biol.* **22**, 986-998.
Yajima, I., Colombo, S., Puig, I., Champeval, D., Kumasaka, M., Belloir, E., Bonaventure, J., Mark, M., Yamamoto, H., Taketo, M. M., et al. (2013). A subpopulation of smooth muscle cells, derived from melanocyte-competent precursors, prevents patent ductus arteriosus. *PLoS ONE* **8**, e53183.
Yajima, I. and Larue, L. (2008). The location of heart melanocytes is specified and the level of pigmentation in the heart may correlate with coat color. *Pigment cell & melanoma research* **21**, 471-476.

Second decision letter

MS ID#: DEVELOP/2020/194407

MS TITLE: Stabilization of ß-catenin promotes melanocyte specification at the expense of the Schwann cell lineage

AUTHORS: Sophie Colombo, Valerie Petit, Roselyne Y Wagner, Delphine Champeval, Ichiro Yajima, Franck Gesbert, Irwin Davidson, Veronique Delmas, Lionel Larue, and Zackie Aktary
ARTICLE TYPE: Research Article

I have now received all the referees reports on the above manuscript, and have reached a decision. The referees' comments are appended below, or you can access them online: please go to BenchPress and click on the 'Manuscripts with Decisions' queue in the Author Area.

The overall evaluation is positive and we would like to publish a revised manuscript in Development. Please attend to the minor issues listed by Referee 3 and then I will be happy to accept your study.

We are aware that you may currently be unable to access the lab to undertake experimental revisions. If it would be helpful, we encourage you to contact us to discuss your revision in greater detail. Please send us a point-by-point response indicating where you are able to address concerns raised (either experimentally or by changes to the text) and where you will not be able to do so within the normal timeframe of a revision. We will then provide further guidance. Please also note that we are happy to extend revision timeframes as necessary.

Reviewer 1

Advance summary and potential significance to field

The authors addressed all my comments, and the paper is ready to be published

Comments for the author

The authors addressed all my comments, and the paper is ready to be published

Reviewer 2

Advance summary and potential significance to field

This manuscript adds to the evidence that there is a "second wave" of melanoblast differentiation, and perhaps puts a limit on the extent of these cells to overall pigmentation.

© 2022. Published by The Company of Biologists under the terms of the Creative Commons Attribution License (https://creativecommons.org/licenses/by/4.0/).

Comments for the author

The authors have addressed my concerns in the revised manuscript

Reviewer 3

Advance summary and potential significance to field
I would like to congratulate the authors for this very nice study, in which they have addressed all questions raised by this reviewer by adding new experiments and adequately clarifying specific points.

Comments for the author

There are only some minor issues that should be taken care of before publication:
- Abstract, lane 55. The sentence “hyperactivation in vitro of the Wnt/ß-catenin pathway repressed FoxD3 expression, which would be necessary for melanocyte development, through Mitf-M activation.” is a bit confusing. While repression of FoxD3 is indeed necessary for melanocyte development, FoxD3 expression would be necessary for Schwann cell development. Since “which” could refer to both repression of FoxD3 or FoxD3 expression, I would rephrase this statement.
- In the reply to this reviewer’s comments, the authors correctly refer to a paper by Hari et al., 2012 (Development), but in the manuscript they cite another paper by these authors from 2002. This should be corrected.
- As proposed, the authors now included a non-pathogenic Schwann cell line in their experimental settings (Fig. 7). However, as this is a non-commercially available line, the authors should reference a paper or include representative images that show that these cells express common Schwann cell markers in vitro.

Second revision

Author response to reviewers' comments

Reviewer 1 Advance summary and potential significance to field

The authors addressed all my comments, and the paper is ready to be published

Reviewer 1 Comments for the author

The authors addressed all my comments, and the paper is ready to be published

We thank the reviewer for this positive comment.

Reviewer 2 Advance summary and potential significance to field

This manuscript adds to the evidence that there is a "second wave" of melanoblast differentiation, and perhaps puts a limit on the extent of these cells to overall pigmentation.

Reviewer 2 Comments for the author

The authors have addressed my concerns in the revised manuscript

We thank the reviewer for this positive comment.

© 2022. Published by The Company of Biologists under the terms of the Creative Commons Attribution License (https://creativecommons.org/licenses/by/4.0/).

Reviewer 3 Advance summary and potential significance to field

I would like to congratulate the authors for this very nice study, in which they have addressed all questions raised by this reviewer by adding new experiments and adequately clarifying specific points.

We thank the reviewer for this positive comment.

Reviewer 3 Comments for the author

There are only some minor issues that should be taken care of before publication:

- Abstract, lane 55. The sentence “hyperactivation in vitro of the Wnt/ß-catenin pathway repressed FoxD3 expression, which would be necessary for melanocyte development, through Mitf-M activation.” is a bit confusing. While repression of FoxD3 is indeed necessary for melanocyte development, FoxD3 expression would be necessary for Schwann cell development. Since “which” could refer to both repression of FoxD3 or FoxD3 expression, I would rephrase this statement.

We modified the sentence to "Furthermore, in vitro hyperactivation of the Wnt/ß-catenin pathway, which is required for melanocyte development, induces activation of Mitf-M, in turn repressing FoxD3 expression". The abstract has now 180 words.

- In the reply to this reviewer’s comments, the authors correctly refer to a paper by Hari et al., 2012 (Development), but in the manuscript they cite another paper by these authors from 2002. This should be corrected.

It is now fixed.

- As proposed, the authors now included a non-pathogenic Schwann cell line in their experimental settings (Fig. 7). However, as this is a non-commercially available line, the authors should reference a paper or include representative images that show that these cells express common Schwann cell markers in vitro.

The reference of IPN 02.3 Schwann cell line was already included in the materials and methods section. We now included this reference in the "results" and "materials and methods" sections.

<u>Third decision letter</u>

MS ID#: DEVELOP/2020/194407

MS TITLE: Stabilization of ß-catenin promotes melanocyte specification at the expense of the Schwann cell lineage

AUTHORS: Sophie Colombo, Valerie Petit, Roselyne Y Wagner, Delphine Champeval, Ichiro Yajima, Franck Gesbert, Irwin Davidson, Veronique Delmas, Lionel Larue, and Zackie Aktary
ARTICLE TYPE: Research Article

I am happy to tell you that your manuscript has been accepted for publication in Development, pending our standard ethics checks.

© 2022. Published by The Company of Biologists under the terms of the Creative Commons Attribution License (https://creativecommons.org/licenses/by/4.0/).
